# Spatial and temporal diffusion-control of dynamic multi-domain self-assembled gels†

**DOI:** 10.1039/d0sc06862d

**Published:** 2021-02-08

**Authors:** Lisa Schlichter, Carmen C. Piras, David K. Smith

**Affiliations:** Department of Chemistry, University of York Heslington York YO10 5DD UK David.smith@york.ac.uk

## Abstract

The dynamic assembly of a pH-responsive low-molecular-weight gelator (LMWG) within the pre-formed matrix of a second LMWG has been achieved *via* diffusion of an acid from a reservoir cut into the gel. Self-assembly of the acid-triggered LMWG as it converts from micellar aggregates at basic pH into gel nanofibers at lower pH values can be both spatially and temporally controlled. The pH-responsive LMWG has an impact on the stiffness of the pre-formed gel in the domains in which it assembles. When low acid concentrations are used, LMWG assembly is transient – after the initial proton diffusion phase, the pH rises and disassembly occurs as the system equilibrates. Re-application of additional acid as ‘fuel’ can then re-assemble the LMWG network. Using glucono-δ-lactone (which slowly hydrolyses to gluconic acid) instead of HCl gives slower, more spatially-restricted assembly, and creates longer-lasting pH gradients within the gel. The presence of an agarose polymer gel network improves the mechanical strength of the gels and appears to slightly enhance the rate of proton diffusion. More sophisticated reservoir shapes can be cut into these more mechanically robust gels, enabling the creation of diffusion waves with different geometries, and hence different patterns of LMWG activation. Multiple reservoirs can be used to create overlapping proton diffusion waves, hence achieving differentiated pH patterns in the gel. Using acid diffusion in this way within gels is an intriguing and powerful way of dynamic patterning. The ability to temporally-evolve spatially-resolved patterns using biocompatible weak acids, and the change in rheological performance of the triggered domains, suggest potential future applications of this strategy in tissue engineering.

## Abstract 2

The assembly of a pH-sensitive LMWG within a pre-formed network of a second LMWG can be achieved by diffusing acids from pre-cut reservoirs, giving rise to patterned gels in which the rheological properties evolve with spatial and temporal control.

## Graphical abstract

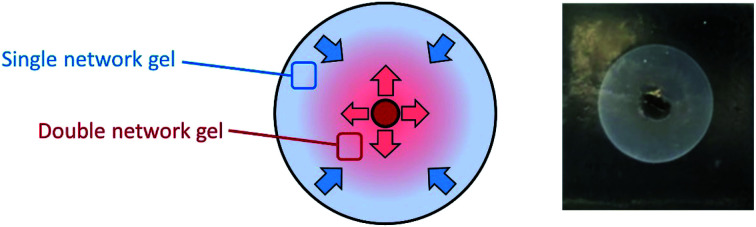

## Introduction

Supramolecular gels are fascinating materials that spontaneously assemble their nanofibrillar networks as a result of non-covalent interactions between low-molecular-weight gelator (LMWG) building blocks.^1^ These gels are highly responsive and tuneable, and are of great interest for use in a wide range of different technological settings.^2^ There has been considerable interest in assembling gels from multiple components as this can be a simple way of formulating additional function into a gel.^3^ In self-sorted ‘multi-gelator gels’, each individual LMWG network can endow the gel with its own unique properties, allowing desired functions to be programmed into the gel in a predictable and controllable way.^4^

Most supramolecular gels are homogenous soft materials, which are relatively weak and unstructured. However, in recent years, increasing efforts have been made to shape, structure or pattern such gels in an attempt to generate highly tuneable materials with advanced applications.^5^ Different techniques have been applied, such as 3D-printing^6^ and photopatterning.^7^ Gels can also be patterned by controlled diffusion. In a key study, Eelkema, van Esch and co-workers demonstrated that by diffusing aldehyde and acylhydrazide components from reservoirs within a preformed polymer gel matrix, self-standing gel objects could be formed in the locations where the two components met and reacted to form an LMWG that subsequently self-assembled with spatial control.^8^ The geometry in which the components were loaded into the gel controlled the resulting pattern. We have also previously explored the diffusion of gelator components across a gel–gel interface to yield interpenetrated two-component gels.^9^

There has been some interest in diffusing acids to generate pH-responsive gels. A diffusing acid has been used to assemble an LMWG – with gel fibrils being aligned with the propagating acidic wave.^10^ Alternatively, polymer cubes can be pre-soaked in HCl and a pH-responsive LMWG on the surface underwent controlled gel assembly.^11^ An acidic catalyst has been patterned on a surface to achieve spatially-directed LMWG assembly.^12^ Localised electrochemical production of H^+^ on an electrode surface has also been used to trigger spatially and temporally resolved LWWG assembly.^13^ Microfluidic methods have also been used to bring an acid trigger into contact with an LMWG with spatial control.^14^ Very recently, Adams and co-workers demonstrated that a photo-acid could create a gradient of acid, leading to a gel with a transient gradient of network assembly, and hence stiffness.^15^

There has recently been increasing interest in gels that assemble in a dynamic way in response to the presence of fuel, and then disassemble, when the fuel is depleted – so-called dissipative self-assembly. In early work, van Esch and co-workers used an alkylating agent as fuel to generate an ester LMWG, while the process was reversed by ambient hydrolysis.^16^ A number of reports built on this, demonstrating hydrogels can exist in non-equilibrium states using a variety of different fuels, and can have their properties reprogrammed over a number of cycles. In recent times this temporal control is increasingly being combined with a degree of spatial resolution.^17^

**Fig. 1:**
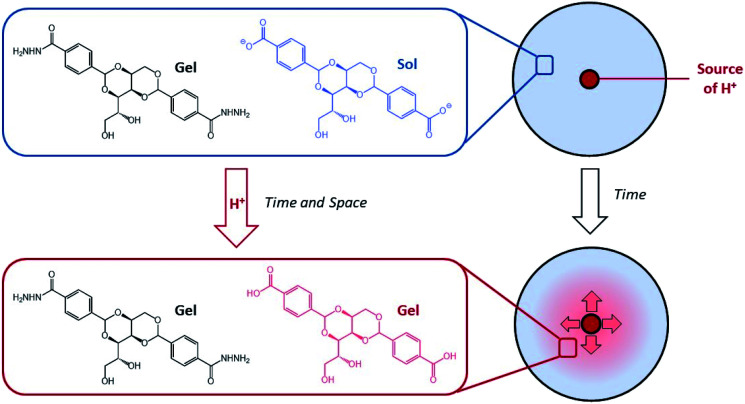
Schematic of H^+^ diffusion experiment, which converts DBS-CO_2_H from its soluble carboxylate form into a self-assembled gel network, within a pre-formed DBS-CONHNH_2_ gel with both spatial and temporal resolution.

We became interested in combining concepts of multi-component gel formation with controlled diffusion to generate gels with spatially-resolved domains having different compositions that exhibit temporal control by dynamically evolving over time in response to proton diffusion. Previously, we reported pH-responsive hydrogels based on an industrially relevant carboxylic acid-modified dibenzylidenesorbitol (DBS-COOH) LMWG,^18^ using slow protonation to form homogeneous gels either by addition of glucono-δ-lactone (GdL), which slowly hydrolyses to release H^+^, or diphenyliodonium nitrate (DPIN), which undergoes photo-induced breakdown and H^+^ release. Recently, we combined this pH-responsive gelator with a thermally responsive, but pH-stable, acylhydrazide-modified dibenzylidenesorbitol (DBS-CONHNH_2_) LMWG hydrogel,^19^ and demonstrated that these two gelators can undergo sequential assembly.^20^ By using DPIN as the proton source, and exposing the sample to UV light under a photo-mask, we achieved localised assembly of DBS-COOH within a pre-formed DBS-CONHNH_2_ gel, to form a patterned multi-domain gel. In such cases, DBS-CONHNH_2_ acts as a supporting matrix to help control the spatial resolution of the self-assembled DBS-COOH network that gets patterned-in. We reasoned that controlled diffusion of an acid through a pre-existing network of DBS-CONHNH_2_ may enable transient and localised assembly of DBS-COOH, creating dynamic multi-domain gels that evolve over time. By loading acid into the DBS-CONHNH_2_ gel at pre-defined locations we planned to achieve spatially and temporally triggered self-assembly of DBS-COOH as the pH drops below the triggering value (Fig. 1). Dynamic gels based on LMWGs that evolve the spatial organisation of their structures and patterns over time are rare. In the longer term, controlling such properties has potential applications in areas such as tissue engineering.

## Results and discussion

### Synthesis and optimisation

Gelators DBS-CONHNH_2_ and DBS-CO_2_H were each synthesised using a simple two-step procedure starting from sorbitol, as previously described by us.^18,19^ Condensation of sorbitol with methyl-4-formylbenzoate gave DBS-CO_2_Me. This compound was then easily converted into DBS-CONHNH_2_ and DBS-CO_2_H by treatment with hydrazine monohydrate and base, respectively. Characterisation of the gelators was in agreement with our previous work.^18,19,21^ These systems form hydrogels as a result of the hydrophobic effect supplemented with intermolecular hydrogen bonding and π–π stacking, with the peripheral substituents playing a key role in modifying solubility and enabling effective gel formation in water. DBS-CONHNH_2_ forms gels *via* a heat-cool cycle which dissolves the LMWG on heating with controlled self-assembly on cooling. DBS-CO_2_H forms gels *via* a pH change, being dissolved in basic conditions in the carboxylate form, and then forming a gel as the pH is lowered below the p*K*_a_ value (5.4) and the carboxylic acid is generated.

### Gel formation and indicator optimisation

To prepare gels, DBS-CO_2_H was dissolved in NaOH(aq) and added to a suspension of DBS-CONHNH_2_ in water. The gelation of the DBS-CONHNH_2_ component was then triggered by a heat-cool cycle. To form gels for the diffusion experiments a basic solution of DBS-CO_2_H and DBS-CONHNH_2_ (each 0.4% wt/vol) was heated and loaded into square Petri dishes (5 × 5 cm), and allowed to cool under ambient conditions. In our initial experiments, a reservoir was then cut in the centre of the gel, and loaded with acid. We chose two different proton sources:

• HCl – as HCl diffuses through the gel, it will acidify the system, leading to DBS-CO_2_H assembly.

• Glucono-δ-lactone (GdL) – this compound must both diffuse through the gel, and hydrolyse to generate H^+^.^22^ Each process will have a different rate, which will modify the dynamics.

To visualize the pH change taking place within the gel, and hence the potential assembly of DBS-CO_2_H, an indicator was used. We tested phenolphthalein, Congo red, Thymol blue and *m*-cresol purple. In all gels a growing ‘acidic’ ring was observed. Phenolphthalein gives very clear colour changes as the acids diffuse, but this occurs at a high pH value that is not particularly useful in terms of indicating the p*K*_a_ value of DBS-CO_2_H. Congo red should usefully indicate in the desired pH range, but we found it did not give clear colour changes in the gel. Thymol blue and *m*-cresol purple were then studied. Both of these indicators can undergo more than one colour change, allowing more detailed understanding of the pH changes occurring within the gel. Visual inspection led us to select Thymol blue as the indicator of choice because its colour changes were clearly differentiated and could be easily discriminated in the gel.

To improve the mechanical properties of the gel, we tested polymer gelator (PG) additives, which are known to be able to form hybrid hydrogels with LMWGs endowing them with greater robustness.^23^ We reasoned this would make it easier to cut well-defined reservoirs into the gels to be loaded with the acids. We tested agarose and calcium alginate as PG additives. The colours of the indicators were brighter in the gels with alginate, making it difficult to see the acid-induced colour change. However, the gels made using agarose as the PG had the same colours as those without. Agarose was therefore selected as the PG for strengthening these gels when required. We have previously demonstrated agarose is compatible with each of DBS-CO_2_H^18,24^ and DBSCONHNH_2_.^25^

**Fig. 2:**
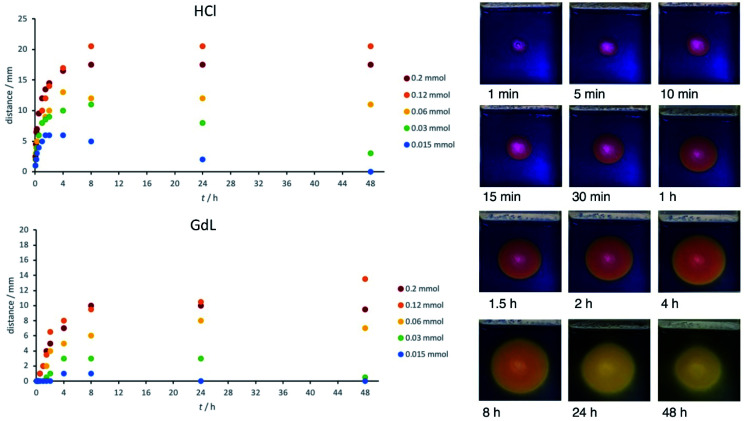
(Top) Graph showing the distance diffused through the gel by HCl, as determined by the visual assembly of DBS-CO_2_H, into an opaque gel over time at different concentrations; (centre) graph showing the distance diffused through the gel by GdL, as determined by the visual assembly of DBS-CO_2_H, over time at different concentrations; (bottom) photographs of the gel over time after addition of HCl (60 μmol) to the central reservoir.

When using Thymol blue as the indicator, as expected, the colour change proceeded outwards in a wave from the central circular reservoir cut into the gel. Interestingly, in addition to the clearly visible colour change, we also observed a difference in the appearance of the gel, which became slightly more opaque. This also occurred in a ring, proceeding outwards from the central reservoir. We ascribe this visual change to the assembly of the DBS-CO_2_H network within the pre-formed DBS-CONHNH_2_ gel as the pH drops below its p*K*_a_ value (for detailed characterisation, see below). This visual change allowed us to estimate the extent of DBS-CO_2_H assembly. Initially, we added HCl to the central reservoir at different loadings. In general, as would be expected, the ring becomes larger when the amount of acid is higher, and the initial diffusion rate out of the reservoir is faster (Fig. 2).

It is important to note, that on its own, a DBS-CONHNH_2_ gel disassembles in the presence of strong acids such as HCl at pH < 2.^19^ To evidence this, we exposed the DBS-CONHNH_2_ gel to HCl at different concentrations (Table S1†). At low HCl loadings, the gel could survive for several hours, but with larger amounts of HCl the gel broke down within an hour. This process is ascribed to protonation of the acylhydrazide group enhancing solubility and giving rise to gel breakdown – we demonstrated this was the case by analysing the acid-exposed gels by ^1^H NMR and MS (Fig. S23 and S24†). Both techniques demonstrated that DBS-CONHNH_2_ remained intact on acid exposure. ^1^H NMR experiments integrated with an internal DMSO standard indicated that >50% of the DBS-CONHNH_2_ disassembled into the mobile, liquid-like phase. We saw no evidence of acetal degradation or scrambling. Interestingly, however, although on their own DBS-CONHNH_2_ gels disassemble in strong acids, in the acid diffusion experiments reported here in this hybrid system, we never saw any evidence of DBS-CONHNH_2_ disassembly or instability (see further discussion and evidence in the NMR characterisation section below). We ascribe the greater stability of the DBS-CONHNH_2_ network here to the presence of DBS-CO_2_^−^, which has a much higher p*K*_a_ value and which the HCl is therefore acting to protonate. In this way, the acid fuel is being used up in assembling the second DBS-CO_2_H network, rather than disassembling the pre-assembled DBS-CONHNH_2_.

### Acid diffusion studies – effect of concentration

Having optimised the system, we went on to study the diffusion of the acid and assembly of DBS-CO_2_H in more detail. In particular, we measured the ‘ring size’ – the distance (radius) to which DBS-CO_2_H appears to form a gel by the formation of a more opaque region. This distance was similar to, but slightly less than, that denoted by the colour change of the indicator in the gel. The radius of apparent gel formation (not the indicator colour change) is plotted in Fig. 2.

When 200 μmol of HCl are present, the acidified ring grows to *ca.* 20 mm over a period of about 8 h, and then remains stable at this size. When 120 μmol of HCl were present, the ring grew slightly larger – it is unclear why this is. When the amount of acid is significantly lower (<60 μmol), the ring grows for a period of time but then starts becoming smaller (Fig. S1†). Indeed, when only 15 μmol of HCl are present, the zone of acidification is completely erased after 48 h. This indicates that the acid diffusing outwards can ultimately be neutralized by the exterior domain, which is basic. The gel can therefore experience a spatially-resolved drop in pH, triggering DBS-CO_2_H assembly, which then is reversed as the pH increases again – indicting the highly dynamic nature of this diffusional approach to gel assembly. This extends Adams and co-workers' previous work exploring the transient nature of gradient mediated assembly in gel systems^15^ and indicates the power of this approach in achieving both spatial and temporal control over gels.

We then loaded GdL at different concentrations into the central well. GdL first has to hydrolyse (timescale, hours) and then once it has done so, is a weak acid, in contrast to the strong acid HCl. These two factors, have a significant impact on the diffusion rate, with initial acidification being much slower (Fig. 2). This is especially true at <60 μmol where there is effectively no acidification for the first few hours. The zone of acidification therefore spreads more slowly, and does not extend as far, typically reaching a distance of *ca.* 10 mm after 24 h (rather than 20 mm). Once again, particularly at lower concentrations, there is evidence that the acidified self-assembled zone decreases in size over extended periods of time as a result of neutralisation as equilibration with the surrounding basic gel occurs (Fig. S2†). In general, it is important to note that the extent of diffusion, and the gradients obtained, can be predicted based on the concentration of acid and the volume of the surrounding matrix.

### Acid diffusion studies – effect of agarose PG

These ‘diffusion ring’ experiments were then repeated in the presence of agarose (Fig. S3 and S4†). The diffusion of acid in the presence of an agarose PG was greater, particularly at intermediate concentrations of HCl (Fig. S5 and S6†). When using GdL in the presence of the agarose PG, the acidified zone increased in size for a longer period of time (up to 48 h), and extended further (>15 mm at 200 μmol GdL) (Fig. S7†). This would suggest that agarose can somehow assist H^+^ transport. The agarose PG also appears to somewhat protect against re-neutralisation of the acidified zone. Interestingly, Hermans, Besenius and co-workers recently reported that proton diffusion through agarose gels appeared to be faster than through other polymer gel networks, possibly as a result of its high polarity^11^ – these results would support that view. There is increasing interest in the diffusion of H^+^ through hydrogels,^26^ and such issues, although of importance in reaction diffusion processes, are not yet fully understood.

### Acid diffusion studies – exact pH determination

**Fig. 3:**
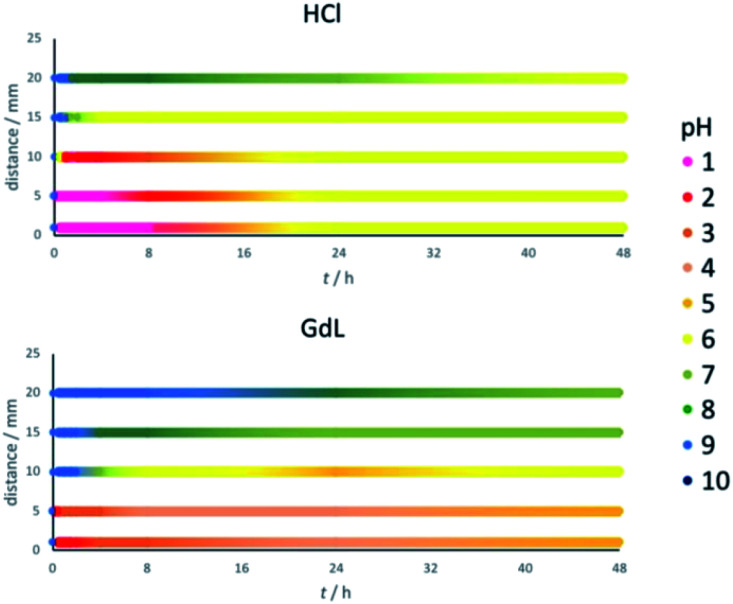
Observed pH values at different distances from the reservoir (distance 0) within the gel and their evolution over time, after adding (top) HCl (120 μmol) (bottom) GdL (120 μmol). The pH values at different distances and times are shown in different colours as indicated in the legend.

Thymol blue has the same colour between pH 2.8 and 8.0. To determine more precise pH values across the gel, pH indicator paper was used at different locations (1, 5, 10, 15 and 20 mm from the reservoir, Fig. 3). Gels were prepared as previously described. Before adding the acid (*t* = 0), the pH value was 9 across the whole gel. On addition of HCl, it rapidly drops to pH 1 at distances up to 5 mm, and pH 2 at 10 mm (Fig. 3, top). The pH drop at longer distances is significantly slower – indeed, pH 6 is only reached after a few hours at 15 mm and after nearly 48 h at 20 mm. As the pH value continues to slowly drop in these more distant regions, in those areas closest to the reservoir the pH value rises somewhat as the system equilibrates. These central regions also eventually end up around pH 6. We therefore suggest that the final pH of the hybrid gel domain in this case is around 6.

Performing the same experiment using GdL as proton source leads, as expected, to smaller and slower changes in pH (Fig. 3, bottom). The central locations at 1 and 5 mm from the reservoir initially drop to a pH of 2, but over the following hours this increases to pH 4/5. At 10 mm from the reservoir, the pH slowly drops to 4/5 over a period of 24 h, and then rises back slightly to pH 6. Further away from the reservoir, at 15 and 20 mm, the pH never drops to values at which the assembly of DBS-CO_2_H would be triggered, ending up at pH 7/8 after 48 h. This is consistent with the earlier results (Fig. 3, bottom), which indicated DBS-CO_2_H only assembled up to a distance of *ca.* 10 mm. Interestingly, after 48 hours, the gel triggered with GdL still contains a significant pH gradient across a 20 mm radius, whereas the HCl system has effectively equilibrated at pH 6. This demonstrates that choice of acid source can have a very significant impact on the dynamics, and temporal properties, of the spatially-resolved assembly.

Repeating the experiments in the presence of agarose (Fig. S8†) showed effectively the same trends for both HCl and GdL diffusion, but once again, in both cases, the processes were faster when agarose is present. A slight pH gradient is still present in the GdL system after 48 h, but it has equilibrated more than in the absence of agarose. Once again, this suggests that a supposedly ‘inert’ PG component can, nonetheless, influence the dynamics of diffusion processes within gels – we note the importance of further studies in this important area.^26^

### Acid diffusion studies – oscillating gels

**Fig. 4:**
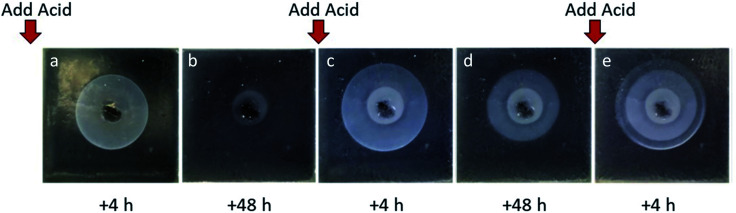
Photographs of gels without indicator (taken against a black background). HCl (30 μmol) was added to the sample three times (*t* = 0, 48 h and 96 h). From left to right, the images show the gel (a) 4 h after the first addition of HCl as the DBS-CO_2_H has reached its maximal radius; (b) 48 h after addition, the system has equilibrated and the DBS-CO_2_H ring has largely disassembled; (c) 4 h after the second addition of HCl, with the second DBS-CO_2_H ring extending beyond the first; (d) 48 h after the second addition as the second DBS-CO_2_H has decreased in size due to equilibration; (e) 4 h after the third addition of HCl, with rings of DBS-CO_2_H associated with the first, second and third waves of acid addition each being clearly visible.

We were particularly interested in the observation that when only small amounts of acid were used, DBS-CO_2_H assembly could be a transient process, being erased as the system eventually comes to equilibrium and the pH value rises. We were therefore interested to know what would happen if additional acid was added to the central reservoir at a defined timepoint. In this case, the acid can be considered as a ‘fuel’ to drive further self-assembly. For the best possible observation of the DBS-CO_2_H ring, no indicator was used during these experiments (Fig. 4). Three cycles of acid addition were performed and in each case a new gel ring was formed. In each cycle of acid addition, the gel ring grew, and then shrank again (Fig. S9–S11†). The second and third rings grew larger than the first, indicative of the fact that a degree of pH lowering is already in place after the earlier cycles of proton-driven assembly and equilibrative disassembly. Comparison of different concentrations of acid indicated that as expected, larger amounts of acid drove the formation of larger transient rings in a predictable way. If GdL was used as the source of protons, transient rings were once again obtained, although in this case they were smaller in agreement with the previous results. In the presence of agarose, larger rings were obtained, once again in agreement with the non-oscillating experiments.

### Acid diffusion studies – spatially-resolved gelation in different patterns

**Fig. 5:**
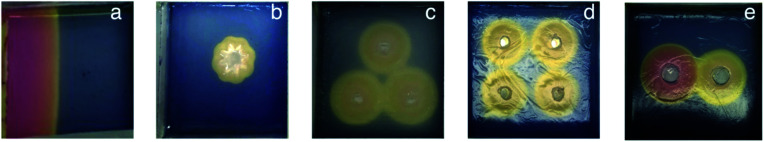
Photographs of gels with different patterns. (a) Gel with a long vertical reservoir down the left hand side of the sample tray 4 h after addition of HCl (170 μmol); (b) gel with a flower-shaped reservoir, 15 min after addition of GdL (170 μmol); (c) gel with three reservoirs arranged in a triangle, 1 h after addition of GdL (170 μmol in each reservoir); (d) gel with four reservoirs arranged in a square, 1.5 h after addition of GdL (170 μmol in each reservoir); (e) gel with two reservoirs, 1 h after addition of HCl (60 μmol) in the left-hand reservoir and GdL (170 μmol) in the right-hand reservoir.

Having characterised and understood the dynamic principles and temporal properties of this gelation system, we were then interested to demonstrate that spatial resolution could be obtained with greater sophistication than a simple circular design. To achieve this, we cut reservoirs of different shapes, or multiple reservoirs, into the gel such that acid could simultaneously diffuse in different directions from different locations (Fig. 5). In order to cut these more sophisticated reservoir patterns into the gel, it was necessary for the gel to have a reasonable degree of mechanical robustness, and for this reason, these studies were only performed on the hybrid gels in which the agarose PG was also present.

When the acid was loaded into a thin vertical reservoir on the left hand side of the tray, linear diffusion in one direction through the gel was obtained (Fig. 5a, S12 and S13†). Diffusion was slightly faster in the centre of the gel tray than at the walls, but in general, wall effects on the diffusion wave were limited.

When the reservoir was cut in the shape of a flower, a polygonal pattern was initially formed, that later became a circle (Fig. 5b, S14 and S15†). This reflects the exposure of different parts of the surrounding matrix to the effective surface area of the cut reservoir.

We then investigated patterns of multiple reservoirs. Three holes arranged in a triangle were cut and filled with acid. Each of the circles became bigger at an equal rate (Fig. 5c, S16 and S17†). After a certain amount of time, the pH value between the reservoirs fell further where the protons of two holes meet each other. In this way, the overlapping diffusion waves from different reservoirs can, in principle, drive different LMWG assembly processes ‘inside’ a pattern than ‘outside’ the pattern.

Finally, two reservoirs were cut in a gel. One was loaded with HCl and the other with GdL. The pH changed more significantly, and more rapidly, around the HCl hole than the GdL hole (Fig. 5e and S20†), as would be expected based on the results presented above. Again, a region of a lower pH value. The same effect was obtained for gels with four reservoirs arranged in a square (Fig. 5d, S18 and S19†). In this way, carefully defined areas of the gel become exposed to lower pH values, which in principle can better build the additional gel network. Between the holes this can be seen – in this case, this region is asymmetric in nature as it is influenced by acids from the two different reservoirs diffusing in different directions. Loading different acid sources in different reservoirs is therefore an effective way of generating asymmetric dynamic patterns.

### Acid diffusion studies – gel without DBS-CO_2_H

Up to this point, we have assumed that the slightly opaque ring that forms in the region where the indicator shows a lower pH value appears because the DBS-CO_2_H assembles and forms a gel, in-line with observations made in the bulk gels, where this hybrid gel is observed to be slightly opaque.^20^ As a key control experiment, we therefore monitored the effect of GdL diffusion on gels in the absence of DBS-CO_2_H (Fig. S21 and S22†). Although the colour of the indicator changed in exactly the same way as described above, as expected, no opaque ring was observed. We therefore confidently assign the opaque ring as being indicative of the self-assembly of DBS-CO_2_H.

### Gel characterisation

Having demonstrated that reservoirs cut into gels can be used to deliver acids into gels and achieve dynamic control over pH and hence DBS-CO_2_H assembly, it was vital that we characterised the gels being formed using this approach to understand this system in as much detail as possible and ensure that the assumptions about the assembly of the DBS-CO_2_H network made above, are correct.

### Gel characterisation – ^1^H NMR

Gelators in the solution phase can be observed by ^1^H NMR, whereas once they have self-assembled into solid-like nanofibers they become immobilised and hence cannot be detected using this technique.^27^ A gel containing both LMWGs, DBS-CO_2_H and DBS-CONHNH_2_, was prepared in the usual way, only on a smaller scale with deuterated solvents. A reservoir was cut in the centre, and HCl(aq) added. Two hours later, aliquots were taken from the inner and outer domains of the gel (Fig. S25†), carefully pipetted into an NMR tube and ^1^H NMR spectra recorded. This procedure may somewhat damage the gel, and lead to disassembly, but was the best compromise we could find for trying to use ^1^H NMR to monitor the spatially-resolved gel experiment ‘*in situ*’. By using a known amount of DMSO as an internal standard, the concentration of bound and unbound DBS-CO_2_H could be calculated. In the outer domain of the gel there was only 3% immobilised DBS-CO_2_H, with 97% being observed in the ^1^H NMR spectrum as the soluble free carboxylate form (Fig. S26†). Conversely, in the inner domain, the gelator was fully assembled and could not be detected by ^1^H NMR (Fig. S27†). In both cases, it was clear that the DBS-CONHNH_2_ network remained intact, with effectively none of the ‘mobile’ gelator being observed in solution. This provides unequivocal evidence that the diffusion of acid through the gel does indeed give rise to the protonation, self-assembly and immobilisation of DBS-CO_2_H in a spatially-defined way, while the pre-formed self-assembled DBS-CONHNH_2_ network maintains its supporting self-assembled role.

When the experiment was performed with GdL added to the reservoir, aliquots of the inner domain were taken over time and analysed by ^1^H NMR. After 1 h, 45% of the DBS-CO_2_H was immobilised, which rose to 87% after 6 h and 97% after 24 h (Fig. S28, S29 and Table S2†). This clearly illustrates the stepwise temporal evolution of the gel as GdL, after initially diffusing quickly into the inner domain, is slowly hydrolysed, lowering the pH and driving the self-assembly of DBS-CO_2_H.

To provide more detailed kinetic insight, gels were prepared in NMR tubes, and the acid sources added on top. NMR spectra were recorded over time (Fig. S30 and S31†). For analysing the overall concentration of bound DBS-CO_2_H in the gel, gels were prepared in NMR tubes and acid was added on top. NMR spectra were recorded overnight. For both HCl and GdL, an increasing percentage of immobilised DBS-CO_2_H was observed over time. As expected, the LMWG assembles more rapidly in the presence of HCl than GdL, and also when the concentration of acid is higher. It should be noted, however, that the narrow NMR tube means diffusion of the acid is very significantly slower, and even after 12 h is incomplete. A further experiment explored assembly after 24 and 48 h. For HCl, immobilisation was almost complete after 48 h and the response was dependent on the acid concentration. The percentage of DBS-CO_2_H immobilised using GdL was smaller, but there was still an increasing trend over time. The experiment was repeated with an indicator present, enabling acid diffusion to be visually observed for 48 h (Fig. S32†). This confirmed that the pH value generated with HCl was lower than in the tubes exposed to GdL. After 8 h, the acid had diffused through a little more than half of the tube. After 48 h, a homogeneous colour (and thus pH value) was obtained with HCl, while there was still a pH gradient with GdL.

### Gel characterisation – infrared (IR) spectroscopy

The most significant infra-red stretches are those of N–H for DBS-CONHNH_2_, and C=O for both, DBS-CO_2_H and DBS-CONHNH_2_. The C=O stretch is particularly distinctive, shifting from 1640 cm^−1^ for the xerogel of DBS-CONHNH_2_ to 1685 cm^−1^ for the two-component gel assembled in the presence of DBS-CO_2_H as previously reported.^20^ Samples of the gels were taken from different locations in the diffusion experiments, dried and analysed by IR (Fig. S33†). The gel near the proton source (inner domain) had a C=O stretch at 1691 cm^−1^. In the outer domain of the gel, the C=O stretch was 1601 cm^−1^. This indicates that the DBS-CO_2_H becomes protonated and self-assembles into a hybrid gel in the inner domain closer to the acid reservoir. The much lower wavenumber C=O stretch in the outer domain can be attributed to the fact that in this part of the gel, the carboxylic acid remains deprotonated in the carboxylate form.

The gels containing agarose were also studied by IR (Fig. S34†). This somewhat complicates matters because agarose has its own IR band at *ca.* 1630 cm^−1^. Nonetheless, once again, differences were observed between samples taken from the inner (1700 cm^−1^) and outer (1617 cm^−1^) domains, in broad agreement with the data above. It can therefore be assumed that DBS-CO_2_H assembly takes place in basically the same way in the presence of agarose.

### Gel characterisation – transmission and scanning electron microscopy (TEM and SEM)

**Fig. 6:**
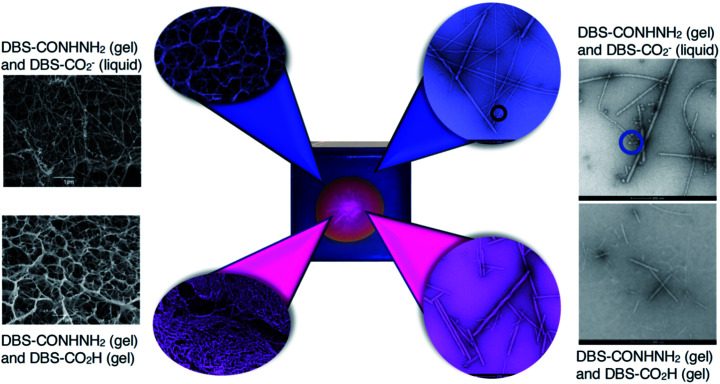
SEM (far left) and TEM (far right) images of control gels prepared in vials with DBS-CONHNH_2_ and DBS-CO_2_H. SEM (left) and TEM (right) of the inner domain (magenta) and the outer domain (blue) of gels formed in trays. In the TEM images of the gel containing solution-phase DBS-CO_2_^−^ (far right top) and the outer domain of the gel (blue), micelles can be visualised (marked with a circle). Scale bars for SEM images = 1 μm, scale bar for TEM images = 200 nm. Further images and enlargements are provided in the ESI.†

Electron microscopy was used to provide insight into the nanoscale structure (Fig. 6 and S35–S44†) of these materials. Obviously, some caution must be applied to the analysis of these data, as the preparation and drying of samples can give rise to artefacts.^28^ However, all gels were handled and treated in the same way in this study, giving some comparative value. Aliquots from the inner and outer domains of a gel created using HCl (or GdL) were analysed by SEM and TEM (Fig. 6 and S35†). We also analysed a number of control samples (Fig. S36†). Initial observations indicated that the gel network is broadly similar in the inner and the outer parts, but there are some subtle differences. In the inner domain, where DBS-CO_2_H is assembled, the average fibre width is 12 nm, with most fibres having diameters < 20 nm (Fig. S38†). However, in the outer domain, where DBS-CO_2_H is present in its soluble carboxylate form, the average fibre width was 29 nm, with most fibres having diameters 20–40 nm (Fig. S39†). Control samples were similar, and similar trends in fibre diameter were also observed in the presence of agarose (Fig. S37, S38 and S40†).

**Fig. 7:**
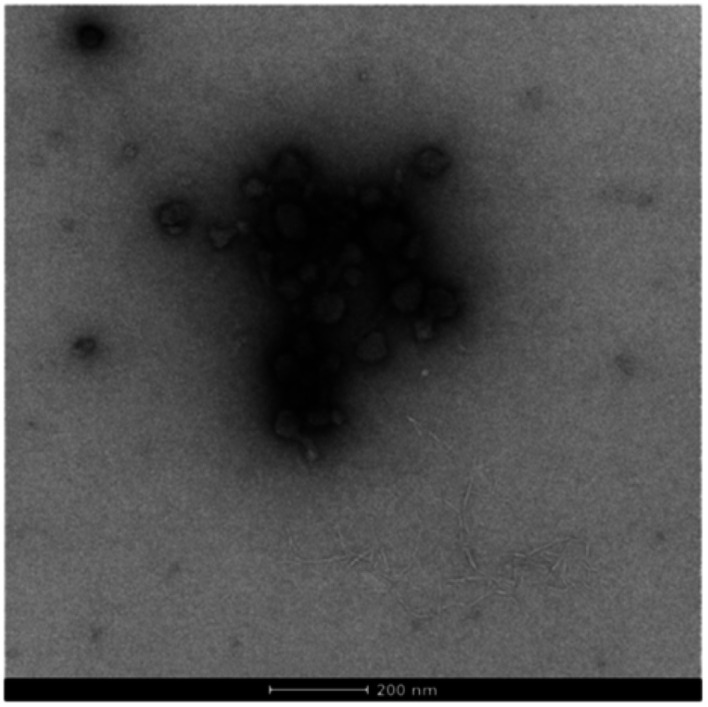
TEM image of micelles formed by DBS-CO_2_^−^ – sample dried from basic conditions, [DBS-CO_2_H] = 9 mM (0.4% wt/vol), scale bar = 200 nm.

More strikingly, in the gel sampled from the outer domain, a second morphology was also observed. This appeared to be spherical in nature with an average diameter of *ca.* 60 nm. We reasoned that the DBS-CO_2_^−^ present in this outer domain, although not forming extended nanofibrillar aggregates responsible for gelation, may assemble into micellar structures as a result of its surfactant-like nature. To test this hypothesis, we prepared a control gel sample which had not been acidified and once again observed the same micelles. Furthermore, TEM of dissolved basic DBS-CO_2_^−^ dried on a TEM grid on its own also clearly indicates the presence of these micelles (Fig. 7), proving the origin of these objects. The fact that these micelles are no longer present in the gel once it has been exposed to acid, provides clear evidence for an assembly mode in which DBS-CO_2_H dissolves in basic conditions to forms micellar aggregates, that subsequently, on exposure to acid, reorganize as the compound becomes protonated, the solubility drops, and it self-assembles instead into gel nanofibers – a process that in this case is being driven by the diffusion of the acid through the supporting DBS-CONHNH_2_ gel.

These observations provide fundamental new insights into the assembly of DBS-CO_2_H and introduce a clear analogy between its assembly mechanism and that of many common gelators from the class of acid-terminated peptides, which are known to form micellar aggregates (usually cylindrical) under basic conditions that then reorganize into gel nanofibres on protonation – a process that has been studied in much detail by Adams and co-workers.^29^

### Characterisation of the gels – rheology

In order to gain important macroscopic insight into the gels, rheological studies were performed using a parallel plate geometry under oscillatory strain. Gels were prepared in circular Petri dishes with a diameter of 8 cm, with HCl being loaded into a central reservoir. Samples were taken from inner and outer domains of the gel (Fig. S45†), carefully transferred to the rheometer and analysed (Fig. S46–S55†). All studies were performed in triplicate and for comparison purposes, control gels made in vials were also analysed.

**Fig. 8:**
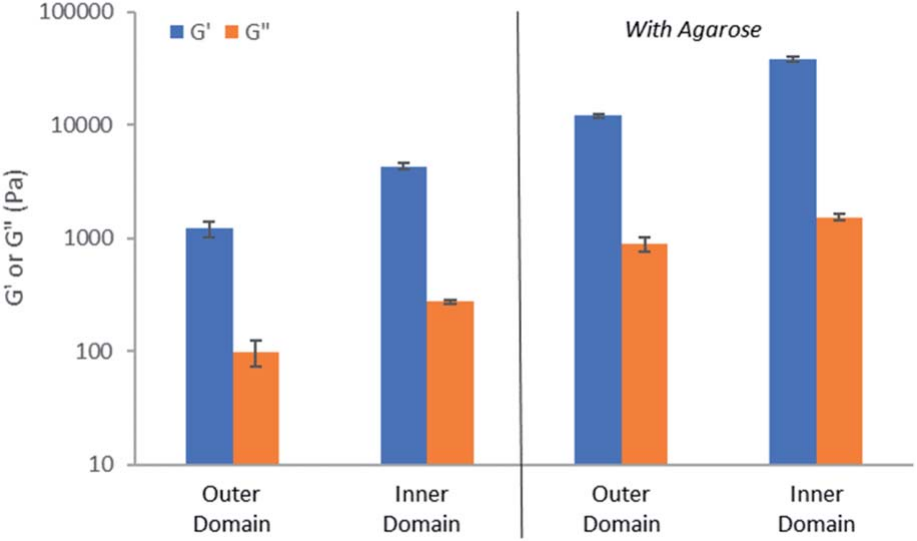
Elastic (*G*′, blue) and viscous (*G*′′, red) moduli of hydrogels sampled from the outer and inner domains, demonstrating the greater stiffness of the inner domain, in which DBS-CO_2_H has assembled into gel-phase nanofibers as a result of the acid stimulus. Data on the left-hand-side is in the absence of agarose, data on the right-hand-side is in the presence of agarose, indicating that agarose stiffens the overall gel.

In all cases, *G*′ was over an order of magnitude larger than *G*′′ (Fig. 8), indicative that all of the samples have gel-like character. Comparing the outer and inner domains of the gel, the *G*′ value increased roughly three-fold, from *ca.* 1200 Pa to 4300 Pa, a significant increase in stiffness resulting from the gel assembly of DBS-CO_2_H in the inner domain within the pre-existing DBS-CONHNH_2_ matrix (Fig. 8). This stiffening effect is in-line with what was previously reported for the sequentially-assembled bulk gels based on these two gelators^20^ and suggests some degree of interaction between the assembled networks, as is often observed for interpenetrated network gels.^30^ In the presence of agarose, the *G*′ value also increased roughly three-fold, from *ca.* 12 100 to 37 900 between the outer and inner domains (Fig. 8), once again demonstrating the stiffening effects of DBS-CO_2_H assembly in the inner domain. The *G*′ values for the gels containing agarose are higher than for those gels without it present, reflecting the enhanced mechanical performance imparted by the PG network.

Control gels had similar rheological properties, with the hybrid gel being significantly stiffer than either of the LMWGs individually. In quantitative terms, the *G*′ and *G*′′ values were different for the control gels, which had been prepared in vials, but this simply reflects the different dimensions of the gel (the gels made in vials are thicker, and are measured with a larger rheometer gap). However, the trends in values are fully consistent with the observations made on the dynamic evolving gels generated in trays in response to proton diffusion.

Importantly, to confirm the temporal control over the assembly of DBS-CO_2_H, samples were taken from the inner domain of the gel at different timepoints, and investigated by rheology (Fig. S56–S58†). Over time, the observed *G*′ value increased from 20 300 to 25 800 Pa, indicating that, as the assembly of DBS-CO_2_H evolves over time in response to the diffusion of protons through the gel, this manifests itself in the rheological performance of the gel, which also exhibits dynamic evolving properties.

These observations provide clear evidence that not only does DBS-CO_2_H assemble in a spatially and temporally controlled manner, but that the dynamic assembly process directly impacts upon the rheological properties of the gel. In this way, the materials performance evolves in a spatially and temporally resolved way that may be harnessed in future applications (see below).

## Conclusions

In summary, we have achieved dynamic assembly of pH-responsive DBS-CO_2_H within a pre-formed matrix of DBS-CONHNH_2_ driven by the diffusion of acid through the gel from reservoirs cut into the gel. We have demonstrated that the assembly of DBS-CO_2_H can be both spatially and temporally controlled depending on the concentration of the acid and have confirmed the formation of a self-assembled DBS-CO_2_H network *via* a wide range of characterisation techniques. In the process of these studies, we found that DBS-CO_2_H forms micellar assemblies at elevated pH, that transition into gel nanofibers as the pH is lowered.

When low acid concentrations are used, the assembly of DBS-CO_2_H is a transient process – after initial acid-induced assembly, the pH subsequently rises and disassembly takes place as the system equilibrates. Reapplication of an acid stimulus as additional ‘fuel’ can then reform the DBS-CO_2_H network. By using different acids, we are able to control the kinetics of the process and the spatial extent of DBS-CO_2_H activation. For example, the use of GdL gives smaller activated domains that form more slowly and exhibit longer lasting pH gradients within the gel.

The presence of an agarose PG network significantly improves the mechanical strength of the gels and also appears to enhance the rate of proton diffusion through the gels. We can cut more sophisticated reservoir patterns into these mechanically robust gels. This can create diffusion waves with different shapes, and hence different spatial patterns of DBS-CO_2_H assembly. We have also used overlapping proton diffusion waves to achieve localised differentiated pH values.

We believe that diffusion processes within gels will be of particular use in creating dynamic and evolving materials for applications such as tissue engineering, in which cell growth can both respond to and direct the gel matrix in which it is taking place.^31^ For example, it is known that stem cells differentiate into different cell types when grown in gels with different stiffnesses.^32^ By achieving spatial and temporal control of the stiffness of a gel, it may be possible to direct stem cell growth in more sophisticated ways than previously achieved. We have previously demonstrated that both DBS-CO_2_H and DBS-CONHNH_2_ can be compatible with cell growth.^4*h*,25^ In the context of the work presented here, there would be a need to start the experiment at lower pH values than 9. This can easily be achieved by pH adjustment of the initial system – in this study we only chose a starting pH of 9 because it matched the requirements of our pH indicator for visualisation of the evolving patterns. Clearly strong acids such as HCl would also be unsuitable for use in a biological setting, but weak acids such as GdL are potentially biocompatible, and could thus diffuse through a gel causing structural and rheological change during the cell growth process by promoting the assembly of a second network, hence influencing biological outcomes in real-time. We suggest that in the future, simple injection of soluble diffusing agents into pH-adjusted gels will be a potential alternative to cutting reservoirs, and could allow the easy loading of gels with different triggers in different locations which can then operate with a high level of spatial and temporal resolution, driven by diffusion processes. Work towards these challenging targets, and their application in a biological setting, are currently in progress in our laboratories.

## Author contributions

The manuscript was written through contributions of all authors. LS performed the experimental work, with guidance and supervision from CCP. DKS envisioned and managed the project and coordinated manuscript writing.

## Conflicts of interest

There are no conflicts to declare.

## Supplementary Material

SC-012-D0SC06862D-s001
