# Supplementary material for: Spatial and temporal diffusion-control of dynamic multi-domain self-assembled gels
Source: Chem Sci. 2021 Feb 8;12(11):4162–72. doi: 10.1039/d0sc06862d (PMC8179439; doi:10.1039/d0sc06862d)
Supplement: SC-012-D0SC06862D-s001 [file SC-012-D0SC06862D-s001.pdf]

Electronic Supplementary Material (ESI) for Chemical Science.
This journal is © The Royal Society of Chemistry 2021

Spatial and Temporal Diffusion-Control of Dynamic Multi-Domain Self-Assembled Gels

Lisa Schlichter,[a] Carmen C. Piras[a] and David K. Smith[a] *

a: Department of Chemistry, University of York, Heslington, York, YO10 5DD, UK.

Email: david.smith@york.ac.uk

SUPPORTING INFORMATION

Contents

S1. General Experimental Methods
S2. Gel Preparation
S3. Stability of DBS-CONHNH$_2$ gel to HCl
S4. NMR Studies
S5. Infrared (IR) Spectroscopy
S6. Transmission and Scanning Electron Microscopy (TEM and SEM)
S7. Rheology
S8. References

S1. General Experimental Methods

All compounds used in synthesis and analysis were purchased from standard commercial suppliers and used as received. The synthesis of DBS-CONHNH$_2$ and DBS-COOH were performed in good yields applying previously reported methods.[1,2] ^{1}H NMR spectra were recorded using a Jeol 400 spectrometer (^{1}H 400 MHz) or a 500 spectrometer (^{1}H 500 MHz). Samples were prepared in D_2O and chemical shifts (δ) are reported in parts per million (ppm). IR spectra of xerogels were recorded on a PerkinElmer Spectrum Two FT-IR spectrometer. TEM images were obtained on a FEI Tecnai 12 G^2 fitted with a CCD camera. Fibre sizes were measured using the *ImageJ* software. SEM images were taken using a JEOL JSM-7600F field emission SEM. Rheology was measured on a Malvern Instruments Kinexus Pro+ Rheometer fitted with a 2 cm parallel plate geometry.

S2. Gel Preparation

S2.1 Acid diffusion studies: DBS-CONHNH$_2$/DBS-COOH hydrogels

DBS-CONHNH$_2$ (0.042 mmol, 0.4 % wt/vol) and DBS-COOH (0.045 mmol, 0.4 % wt/vol) were suspended in water (5 mL total volume) in the presence of thymol blue as an indicator (20 µL of a 1.0 % wt/vol solution in EtOH). NaOH 0.5 M (300 µL) was added to dissolve DBS-COOH; the mixture was sonicated to help the dispersion of the solid particles and then heated until complete dissolution of DBS-CONHNH$_2$. The hot solution was then transferred into a 5x5 cm square tray and left undisturbed to cool overnight, allowing gel formation. Once the gels were formed (blue colour; pH 9-10), a hole was cut in the middle of the tray using a 200 µL pipette tip. HCl or glucono-δ-lactone (GdL) was added into the resulting reservoir (0.2, 0.12, 0.06, 0.03 and 0.015 mmol) giving a gel colour change in correspondence of the proton diffusion ring. The radius of apparent DBS-COOH gel formation (opaque region, not the indicator colour change) was measured with a ruler at regular time intervals (1 min, 10 min, 15 min, 30 min, 1 h, 1.5 h, 2 h, 4 h, 8 h, 24 h and 48 h).

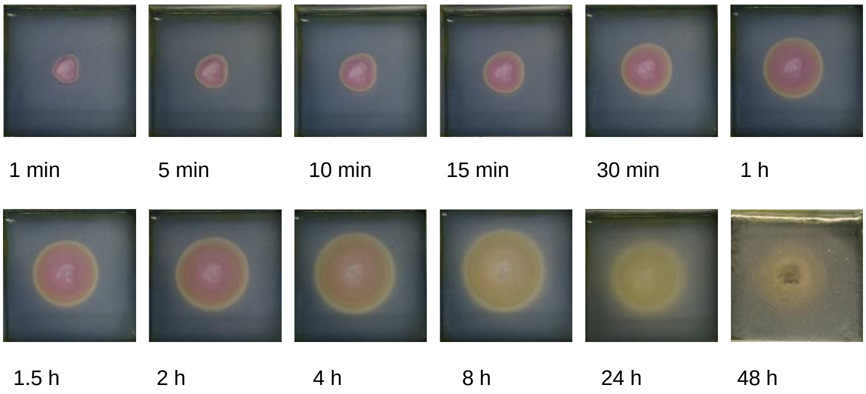

Fig. S1. Photographs of the DBS-CONHNH$_2$/DBS-COOH gel over time, after addition of HCl (0.03 mmol) to the central reservoir.

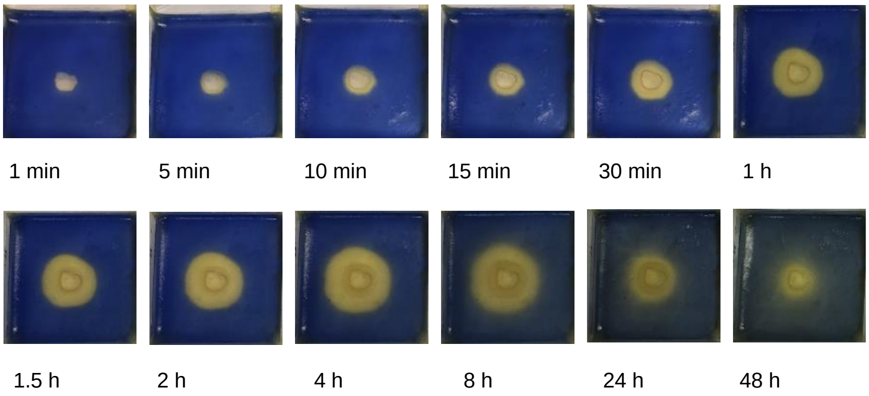

Fig. S2. Photographs of the DBS-CONHNH$_2$/ DBS-COOH gel over time, after addition of GdL (0.03 mmol) to the central reservoir.

S2.2 Acid diffusion studies: DBS-CONHNH$_2$/agarose/DBS-COOH multicomponent hydrogels

DBS-CONHNH$_2$ (0.042 mmol, 0.4 % wt/vol), agarose (1.0 % wt/vol) and DBS-COOH (0.045 mmol, 0.4 % wt/vol) were suspended in water (5 mL total volume) in the presence of thymol blue as an indicator (20 µL of a 1.0 % wt/vol solution in EtOH). NaOH 0.5 M (300 µL) was added to dissolve DBS-COOH; the mixture was sonicated to help the dispersion of the solid particles and then heated until complete dissolution of the gelators. The hot solution was then transferred into a 5x5 cm square tray and left undisturbed to cool overnight, allowing gel formation. Once the gels were formed (blue colour; pH 9-10), a hole was cut in the middle of the tray using a 200 µL pipette tip. HCl or glucono-δ-lactone (GdL) was added into the resulting reservoir (0.2, 0.12, 0.06, 0.03 and 0.015 mmol) giving a gel colour change in correspondence of the proton diffusion ring. The radius of apparent DBS-COOH gel formation (opaque region, not the indicator colour change) was measured with a ruler at regular time intervals (1 min, 10 min, 15 min, 30 min, 1 h, 1.5 h, 2 h, 4 h, 8 h, 24 h and 48 h).

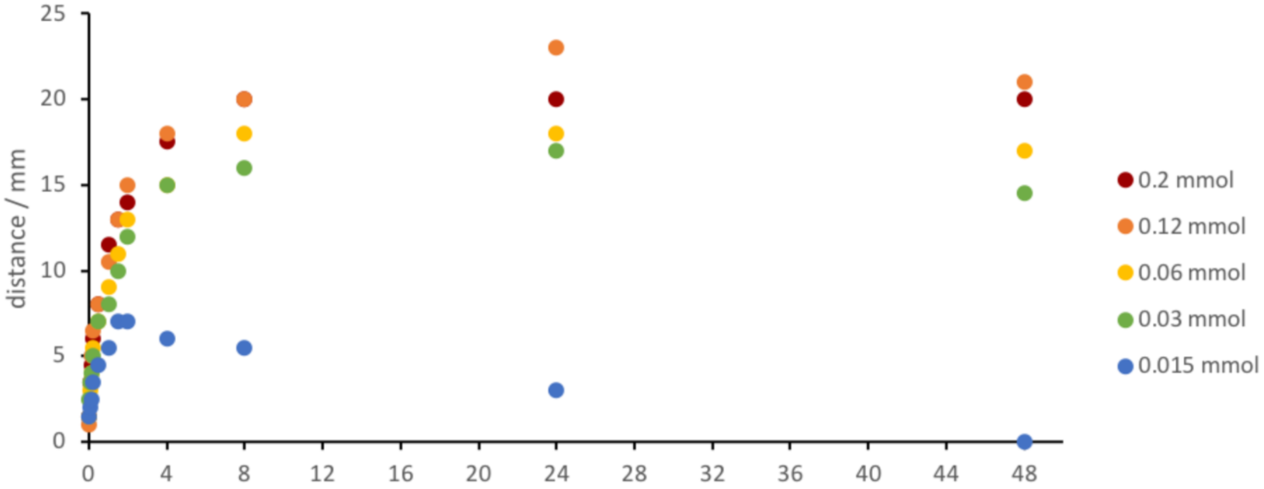

Fig. S3 Graph showing the distance diffused through the DBS-CONHNH$_2$/DBS-CO$_2$H/Agarose gel by HCl, as determined by the visual assembly of DBS-CO$_2$H, into an opaque gel over time at different concentrations.

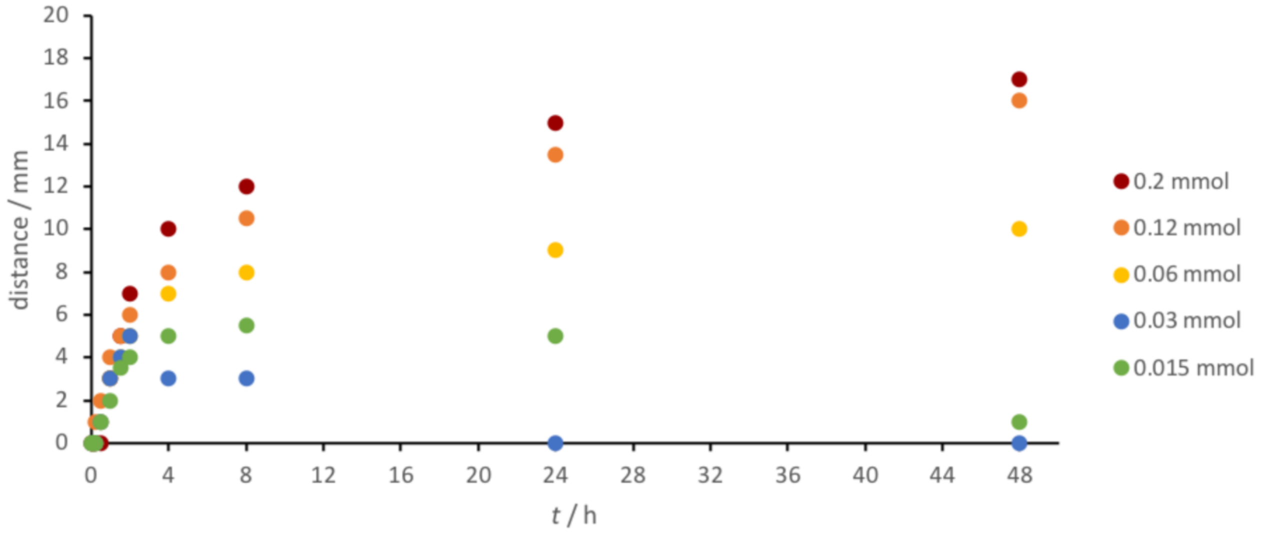

Fig. S4 Graph showing the distance diffused through the DBS-CONHNH$_2$/DBS-CO$_2$H/Agarose gel by GdL, as determined by the visual assembly of DBS-CO$_2$H, over time at different concentrations.

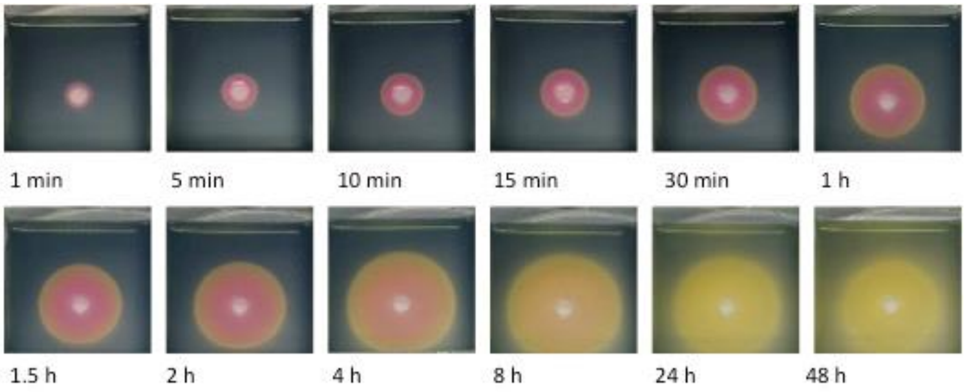

Fig. S5. Photographs of the DBS-CONHNH$_2$/agarose/DBS-COOH gel over time, after addition of HCl (0.06 mmol) to the central reservoir.

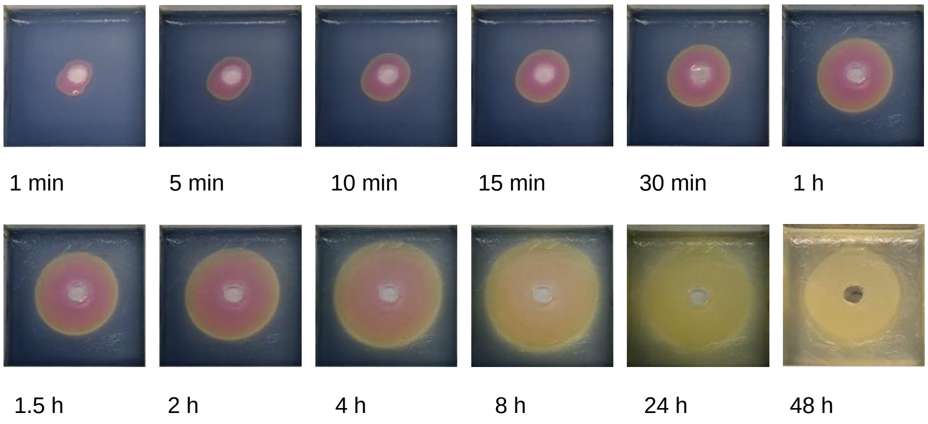

Fig. S6. Photographs of the DBS-CONHNH$_2$/agarose/DBS-COOH gel over time, after addition of HCl (0.03 mmol) to the central reservoir.

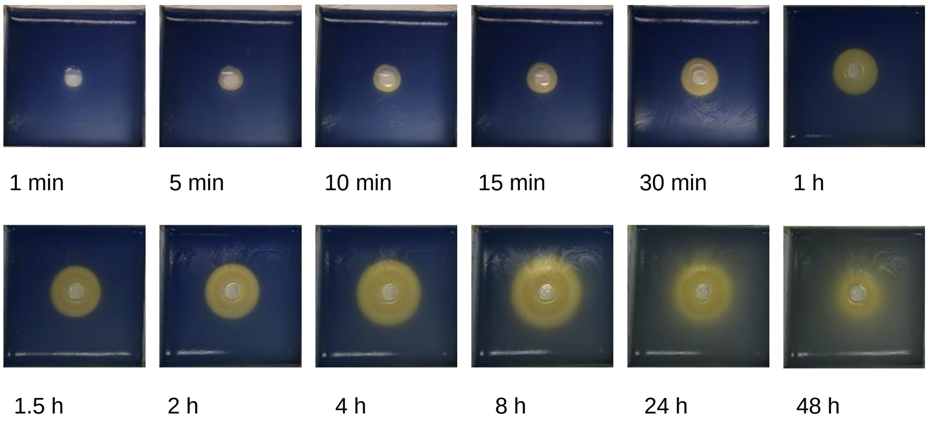

Fig. S7. Photographs of the DBS-CONHNH$_2$/agarose/DBS-COOH gel over time, after addition of GdL (0.03 mmol) to the central reservoir.

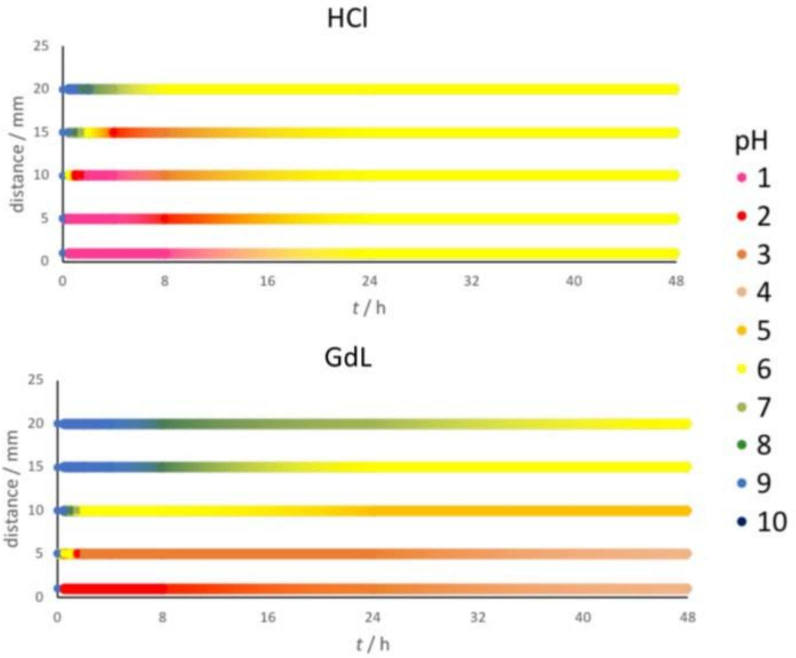

Fig. S8. Observed pH values at different distances from the reservoir (distance 0) within the DBS-CONHNH$_2$/DBSCO$_2$H/Agarose gel and their evolution over time, after adding (top) HCl (120 μmol) (bottom) GdL (120 μmol). The pH values at different distances and times are shown in different colours as indicated in the legend

S2.3 Acid diffusion studies: oscillating gels

DBS-CONHNH$_2$ (0.042 mmol, 0.4 % wt/vol), DBS-COOH (0.045 mmol, 0.4 % wt/vol) and agarose (1.0 % wt/vol, when added) were suspended in water (5 mL total volume). NaOH 0.5 M (300 μL) was added to dissolve DBS-COOH and the mixture was sonicated to help the dispersion of the solid particles and subsequently heated until complete dissolution of the gelators. The hot solution was then transferred into a 5x5 cm square tray and left undisturbed to cool overnight, allowing gel formation. Once the gels were formed, a hole was cut in the middle of the tray using a 200 μL pipette tip. HCl or glucono-δ-lactone (GdL) was added into the resulting reservoir (0.03 and 0.015 mmol) giving an opaque ring in correspondence of the DBS-COOH gel formation. Acid addition was repeated in the same quantities after 48 and 96 h.

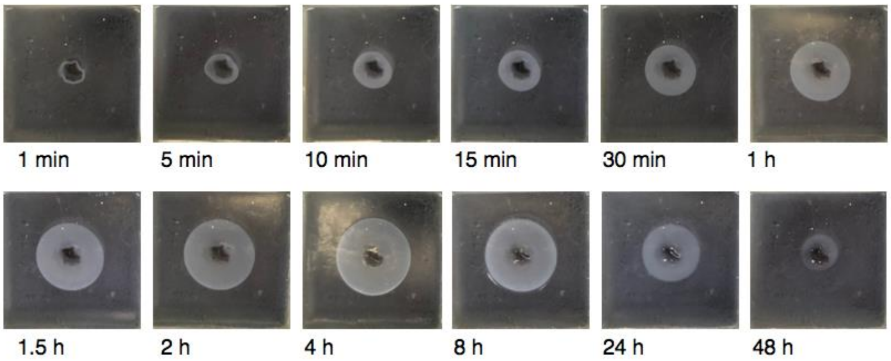

Fig. S9. Photographs of DBS-CONHNH$_2$/DBS-COOH gel without indicator (taken against a black background) after the first addition of HCl (30 μmol) at time 0.

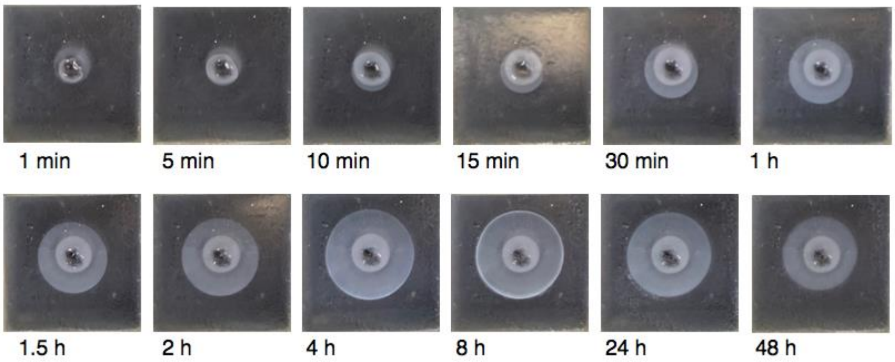

Fig. S10. Photographs of DBS-CONHNH$_2$/DBS-COOH gel without indicator (taken against a black background) after the second addition of HCl (30 μmol), 48 h from the first acid addition.

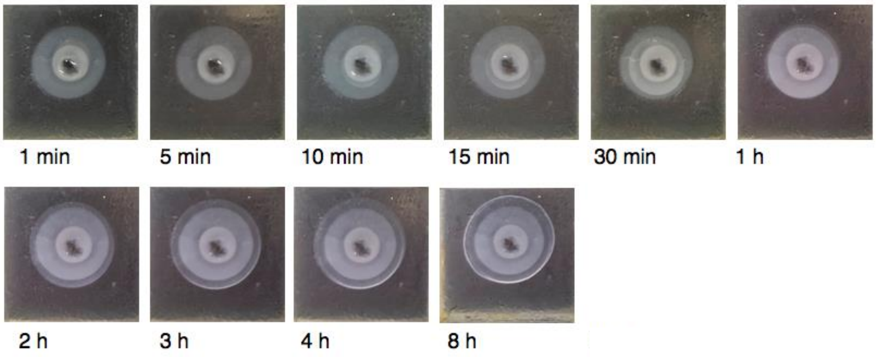

Fig. S11. Photographs of DBS-CONHNH$_2$/DBS-COOH gel without indicator (taken against a black background) after the third addition of HCl (30 μmol), 96 h from the first acid addition.

S2.4 Acid diffusion studies: Spatially-Resolved Gelation in Different Patterns

DBS-CONHNH$_2$ (0.042 mmol, 0.4 % wt/vol), agarose (1.0 % wt/vol) and DBS-COOH (0.045 mmol, 0.4 % wt/vol) were suspended in water (5 mL total volume) in the presence of thymol blue as an indicator (20 µL of a 1.0 % wt/vol solution in EtOH). NaOH 0.5 M (300 μL) was added to dissolve DBS-COOH; the mixture was sonicated to help the dispersion of the solid particles and then heated until complete dissolution of the gelators. The hot solution was then transferred into a 5x5 cm square tray and left undisturbed to cool

overnight, allowing gel formation. Once the gels were formed (blue colour; pH 9-10), the following patterns were cut into the gels: a long vertical line, a flower or multiple holes. HCl or glucono-δ-lactone (GdL) was added into the resulting reservoirs in the specified quantities for the different patterns, giving a gel colour change in correspondence of the proton diffusion wave. Pictures of the gels were taken at regular time intervals (1 min, 5 min, 10 min, 15 min, 30 mins, 1 h, 1.5h, 2 h, 4 h, 8 h, 24 h and 48 h).

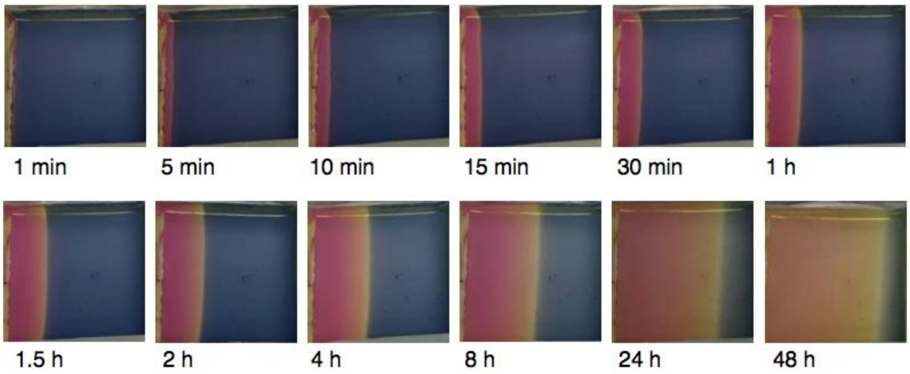

Fig. S12. Photographs of DBS-CONHNH$_2$/agarose/DBS-COOH gel with a long vertical reservoir down the left hand side of the sample tray over time after addition of HCl (0.17 mmol).

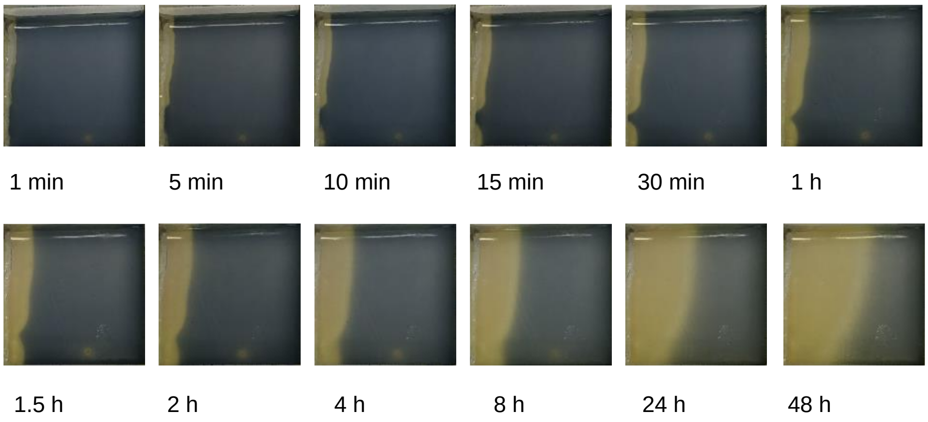

Fig. S13. Photographs of DBS-CONHNH$_2$/agarose/DBS-COOH gel with a long vertical reservoir down the left hand side of the sample tray over time after addition of GdL (0.17 mmol).

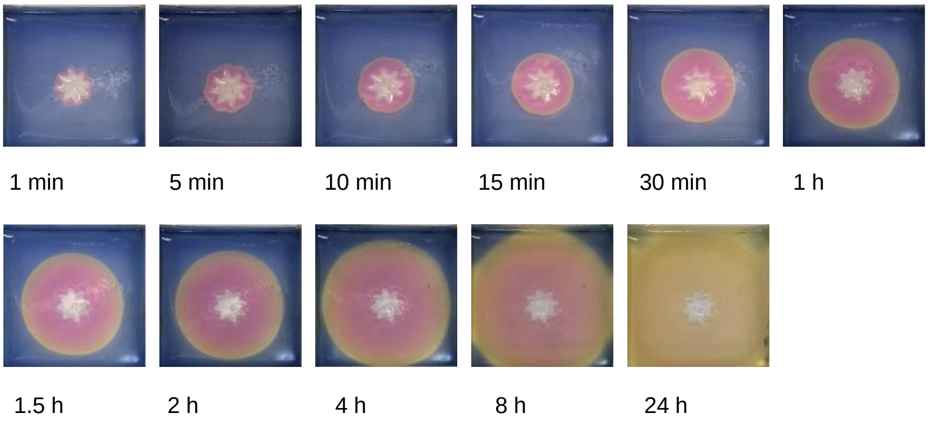

Fig. S14. Photographs of DBS-CONHNH$_2$/agarose/DBS-COOH gel with a flower-shaped reservoir, over time after addition of HCl (0.17 mmol).

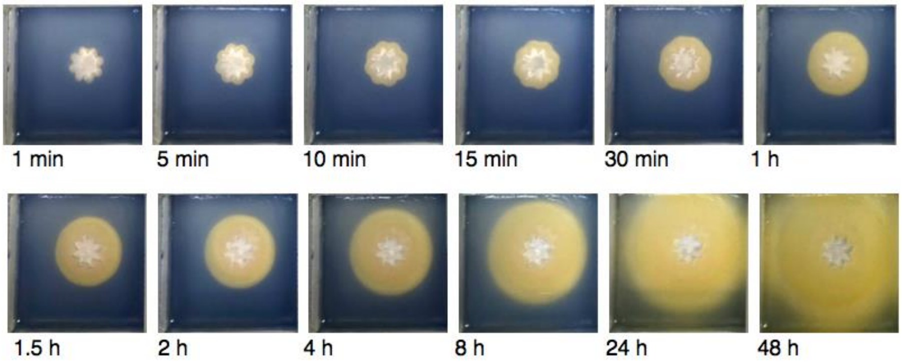

Fig. S15. Photographs of DBS-CONHNH$_2$/agarose/DBS-COOH gel with a flower-shaped reservoir, over time after addition of GdL (0.17 mmol).

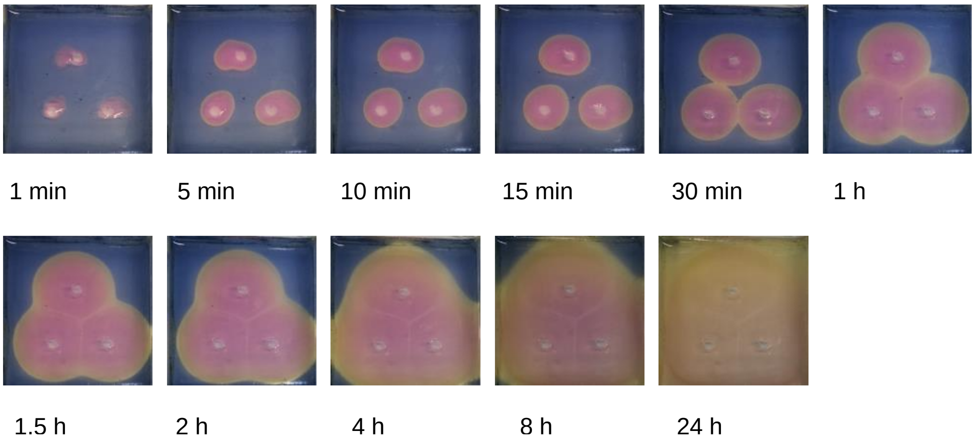

Fig. S16. Photographs of DBS-CONHNH$_2$/agarose/DBS-COOH gel with three reservoirs arranged in a triangle, over time after addition of HCl (0.06 mmol in each reservoir).

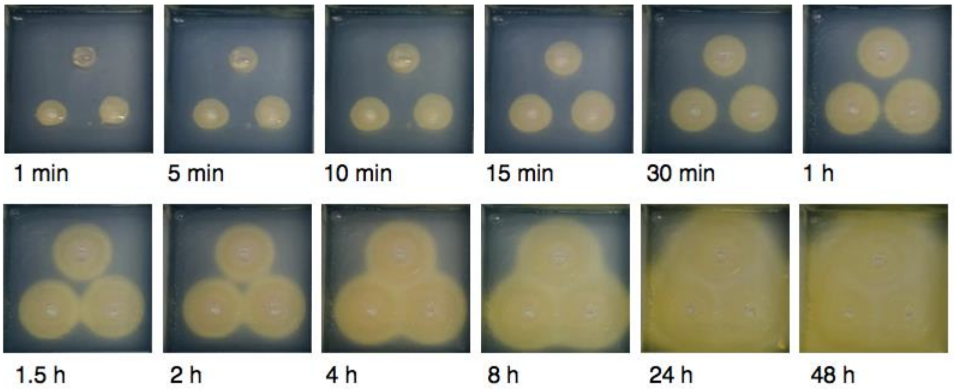

Fig. S17. Photographs of DBS-CONHNH$_2$/agarose/DBS-COOH gel with three reservoirs arranged in a triangle, over time after addition of GdL (0.06 mmol in each reservoir).

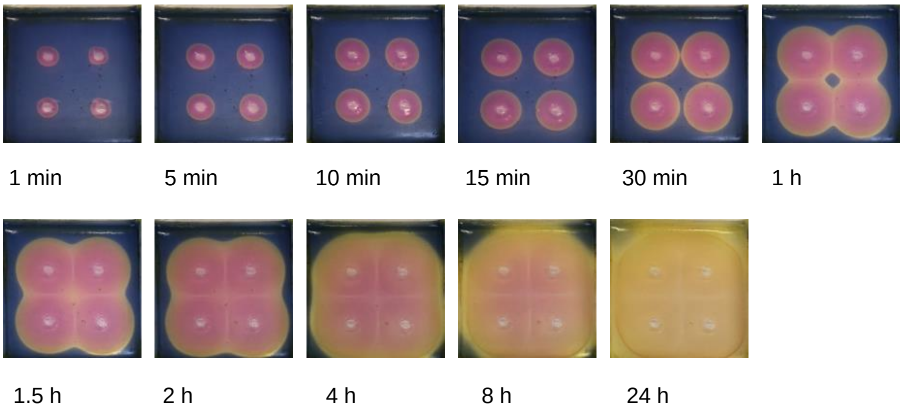

Fig. S18. Photographs of gel with four reservoirs arranged in a square, over time after addition of HCl (0.04 mmol in each reservoir).

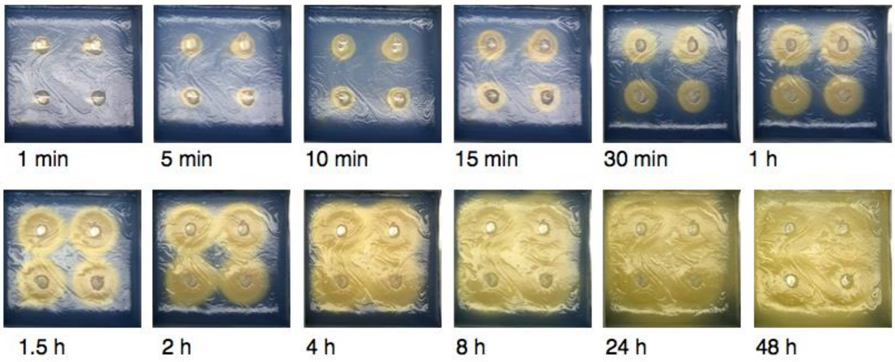

Fig. S19. Photographs of gel with four reservoirs arranged in a square, over time after addition of GdL (0.04 mmol in each reservoir).

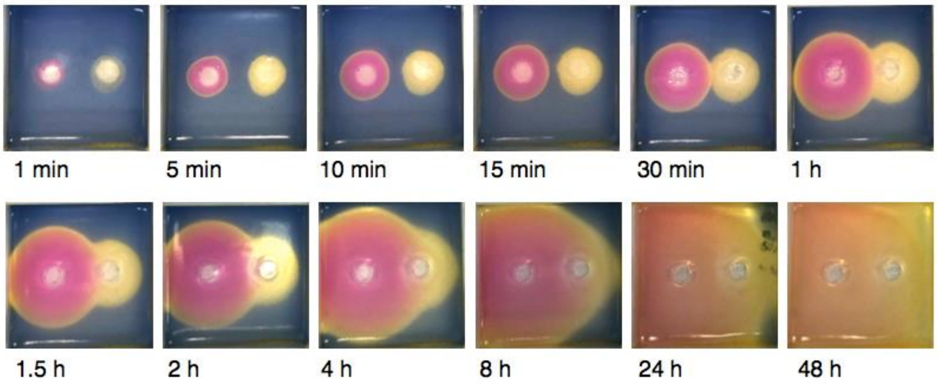

Fig. S20. Photographs of gel with two reservoirs, over time after addition of HCl (0.03 μmol) in the left-hand reservoir and GdL (0.17 mmol) in the right-hand reservoir.

S2.5 Acid diffusion studies: Gels without DBS-COOH

DBS-CONHNH$_2$ (0.042 mmol, 0.4 % wt/vol) and agarose (1.0 % wt/vol) were suspended in water (5 mL total volume) in the presence of thymol blue as an indicator (20 μL of a 1.0 % wt/vol solution in EtOH). The mixture was sonicated to help the dispersion of the solid particles and then heated until complete dissolution of the gelators. The hot solution was then transferred into a 5x5 cm square tray and left

undisturbed to cool overnight, allowing gel formation. Once the gels were formed, a hole was cut in the middle of the tray using a 200 μL pipette tip. HCl or glucono-δ-lactone (GdL) was added into the resulting reservoir giving a gel colour change in correspondence of the proton diffusion ring. Pictures were taken at regular time intervals (1 min, 10 min, 15 min, 30 min, 1 h, 1.5 h, 2 h, 4 h, 8 h, 24 h and 48 h).

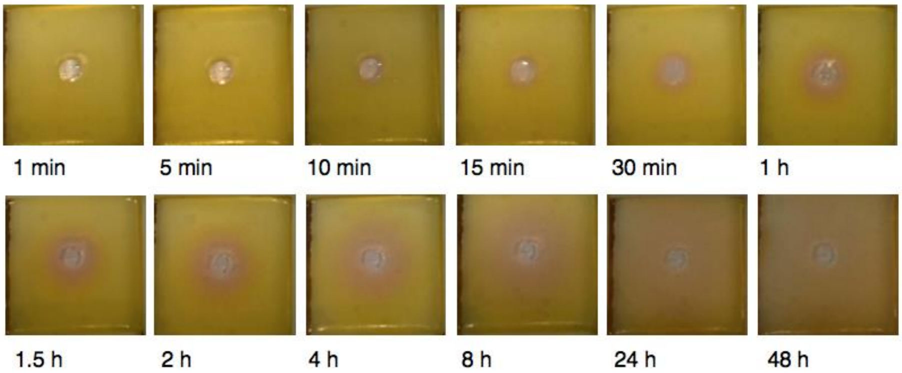

Fig. S21. Photographs of DBS-CONHNH$_2$/agarose gel without DBS-COOH after addition of GdL (0.06 mmol, 75 μL) to the reservoir in the middle.

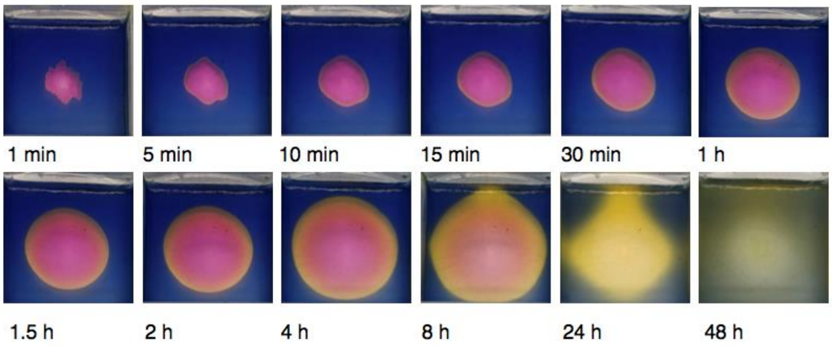

Fig. S22. Photographs of DBS-CONHNH$_2$ gel without DBS-COOH after addition of GdL (0.06 mmol, 75 μL) to the reservoir in the middle. These gels were prepared in the presence of NaOH (300 μL) to reproduce the gel preparation method in the presence of DBS-COOH.

S3. Stability of DBS-CONHNH$_2$ gel to HCl

S3.1 Disassembly of DBS-CONHNH$_2$ in the presence of HCl.

All the gels were prepared in 1 mL volume of water, by heat-cool cycle using a 0.4% wt/vol concentration of DBS-CONHNH$_2$. Different HCl amounts (0.2, 0.12, 0.06, 0.03 and 0.015 mmoles in 100 μL volume) were added on top of each gel and the gel stability was checked over time by visual observation. The pH of the gels was measured before and after acid addition and ESI-MS spectra were recorded before and after acid addition (see example Fig. S23).

Table S1. Visual observations and pH measurements over time after acid addition (PG = partial gel).

HCl added to gels (mmoles)	Before acid addition		After 1.5h		After 3h		After 5h		After 8h		After 24h	
	Visual Observations (left) and pH (right)		Visual Observations (left) and pH (right)		Visual Observations (left) and pH (right)		Visual Observations (left) and pH (right)		Visual Observations (left) and pH (right)		Visual Observations (left) and pH (right)	
0	Gel	8.1	Gel	8.2	Gel	8.1	Gel	8.3	Gel	8.2	Gel	8.5
0.2	Gel	8.2	Liquid	1.2	Liquid	1.3	Liquid	1.2	Liquid	1.2	Liquid	1.1
0.12	Gel	8.1	Liquid	1.3	Liquid	1.2	Liquid	1.3	Liquid	1.3	Liquid	1.4
0.06	Gel	8.6	Liquid	1.5	Liquid	1.5	Liquid	1.3	Liquid	1.3	Liquid	1.3
0.03	Gel	8.0	PG	1.7	Liquid	1.6	Liquid	1.8	Liquid	1.6	Liquid	1.7
0.015	Gel	8.2	Gel	2.2	Gel	2.0	PG	2.1	Liquid	2.2	Liquid	2.2

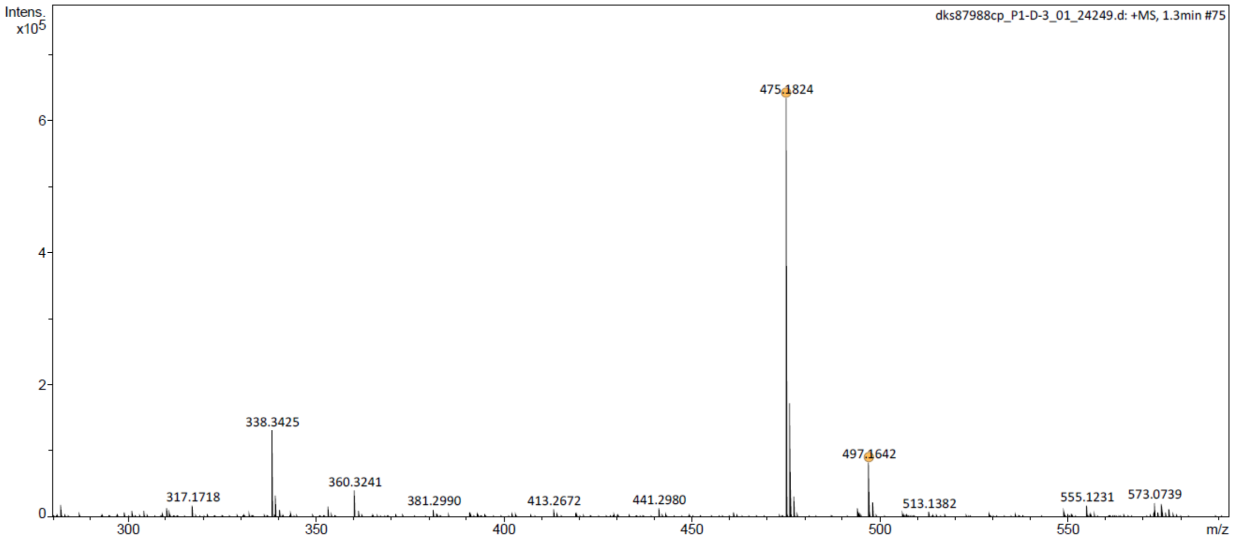

Meas. m/z	#	Ion Formula	m/z	err [ppm]	err [mDa]	mSigma	Mean err [ppm]
475.1824	1	C22H27N4O8	475.1823	-0.2	-0.1	6.7	-1.0
497.1642	1	C22H26N4NaO8	497.1643	0.1	0.0	7.3	3.0

Fig. S23. Mass spectrum of a DBS-CONHNH$_2$ gel 5 h after the addition of HCl (0.2 mmol), demonstrating that DBS-CONHNH$_2$ remains intact.

S3.2 Quantification of disassembly of DBS-CONHNH$_2$ in the presence of HCl

DBS-CONHNH$_2$ (0.4 % wt/vol; i.e. 2.8 mg in 0.7 mL) was dispersed in D_2O (0.7 mL) and sonicated to help the dispersion of the solid particles. DMSO (1.4 μL) was added as an internal standard. The sample was heated until complete dissolution of the solid and the hot solution was then transferred into a NMR tube. The sample was left undisturbed to allow gel formation. Once a gel was formed, HCl (0.14 mmol in 20 μL) was added on top of the gel and left to diffuse through it for 24 hours. After 24h, the ^{1}H NMR was recorded.

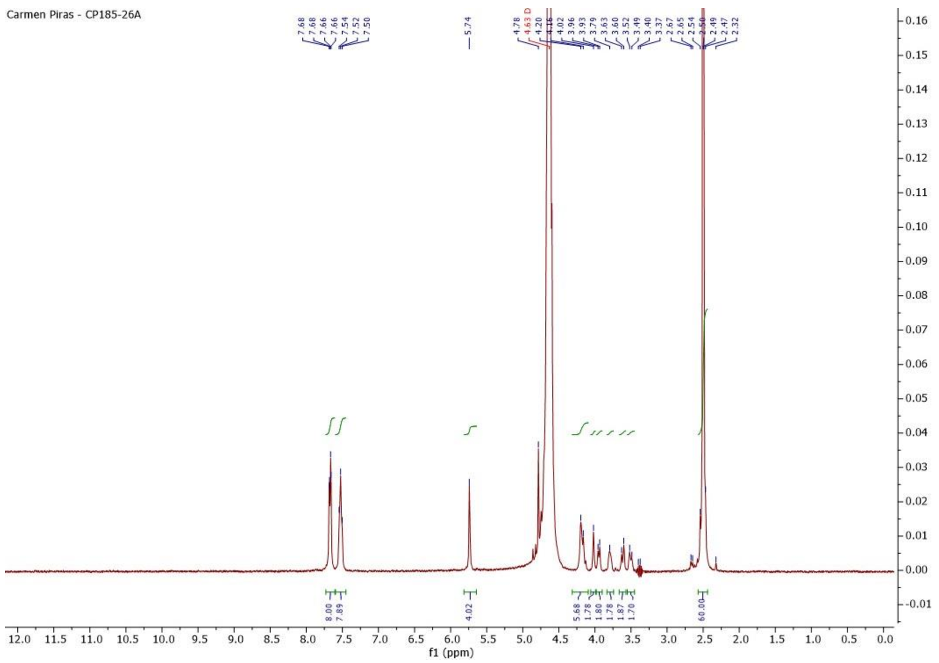

Fig. S24. ^1H NMR of a DBS-CONHNH$_2$ gel 24 h after the addition of HCl (0.14 mmol), demonstrating the release of protonated, but otherwise unmodified, DBS-CONHNH$_2$ into mobile liquid-like solution.

S4. NMR studies

S4.1 Quantification of self-assembled DBS-COOH by ^1H NMR

^1H NMR spectroscopy was used to calculate the percentage of self-assembled DBS-COOH in the DBS-CONHNH$_2$/DBS-COOH gels. The gels used in this experiment were prepared in small petri dishes using deuterated water and deuterated NaOH. DBS-CONHNH$_2$ (0.017 mmol, 0.4% wt/vol) was combined with DBS-COOH (0.018 mmol, 0.4% wt/vol) and NaOD (0.5M, 120 μL) in D_2O (2 mL total volume). The resulting suspension was sonicated and heated until complete dissolution of the solid particles. The hot solution was then transferred to a petri dish (3 cm diameter) and left undisturbed overnight to allow gel formation. Once the gel was formed, an acid reservoir was cut in the middle of gel using a 200 μL pipette tip and DCl (0.012 mmol) or GdL (0.07 mmol) was added and left to diffuse over time. At specific time points (1 h for the DCl gels and 1, 3, 6, 24 h for the GdL gels), 100 μL aliquots were taken from the inner and outer domains of the gel. These were suspended in D_2O (600 μL) and transferred into a NMR tube. DMSO (1.4 μL) was added as an internal standard. The concentration of the mobile components was calculated by comparison of the integrals of relevant peaks (DBS-COOH aromatic peak δ = 7.67) to that of DMSO (δ = 2.50 ppm).

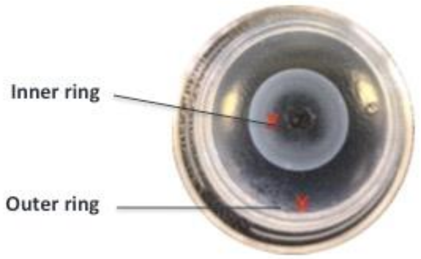

Fig. S25. Photograph of DBS-CONHNH$_2$/DBS-COOH gel in a petri dish after addition of DCl (0.012 mmol) to the reservoir in the middle. Aliquots were taken from the marked regions.

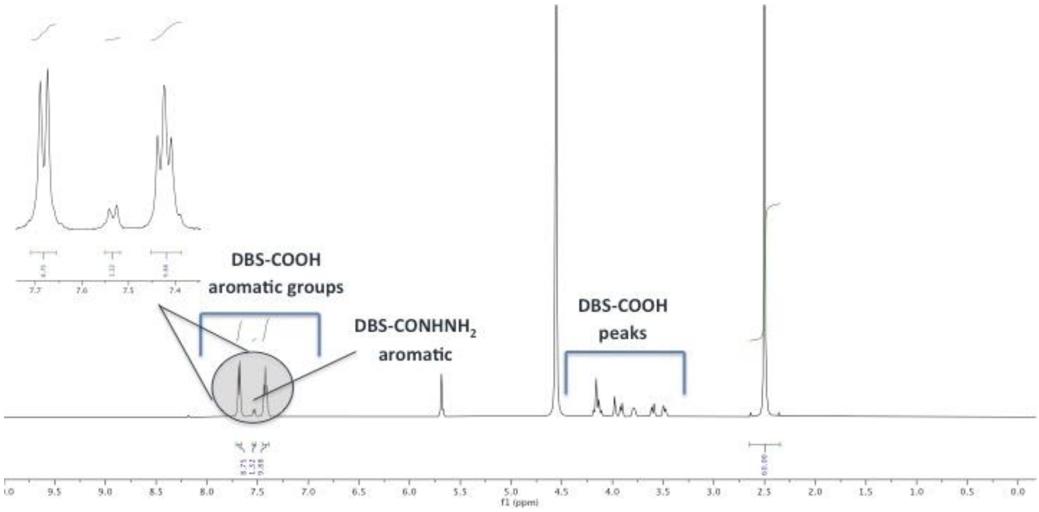

Fig. S26. ^{1}H NMR of outer domain of DBS-CONHNH$_2$/DBS-COOH gel prepared in a petri dish, 1 h after the addition of DCl (0.012 mmol) to the reservoir in the middle.

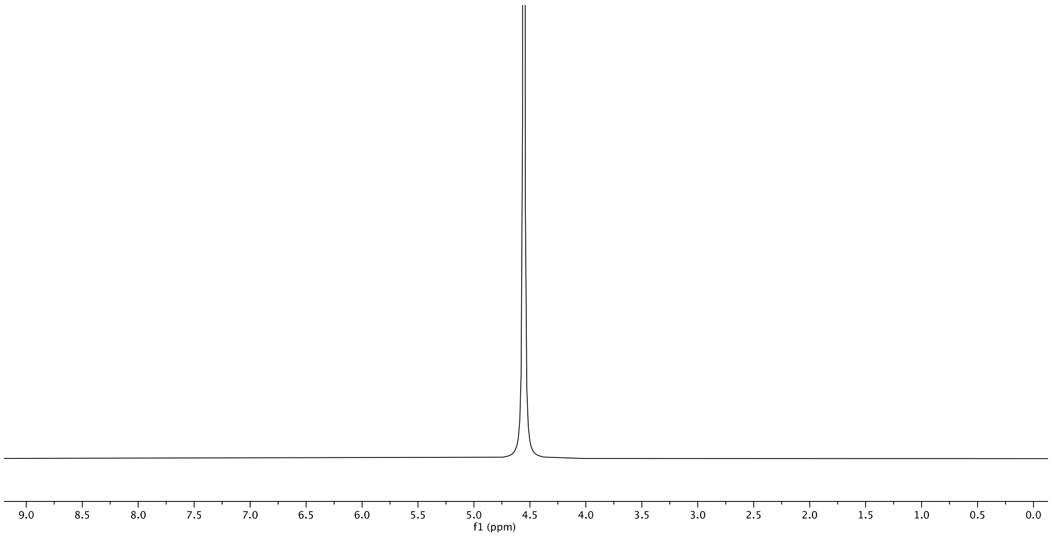

Fig. S27. ^{1}H NMR of inner domain of DBS-CONHNH$_2$/DBS-COOH gel prepared in a petri dish, 1h after the addition of DCl (0.012 mmol) to the reservoir in the middle.

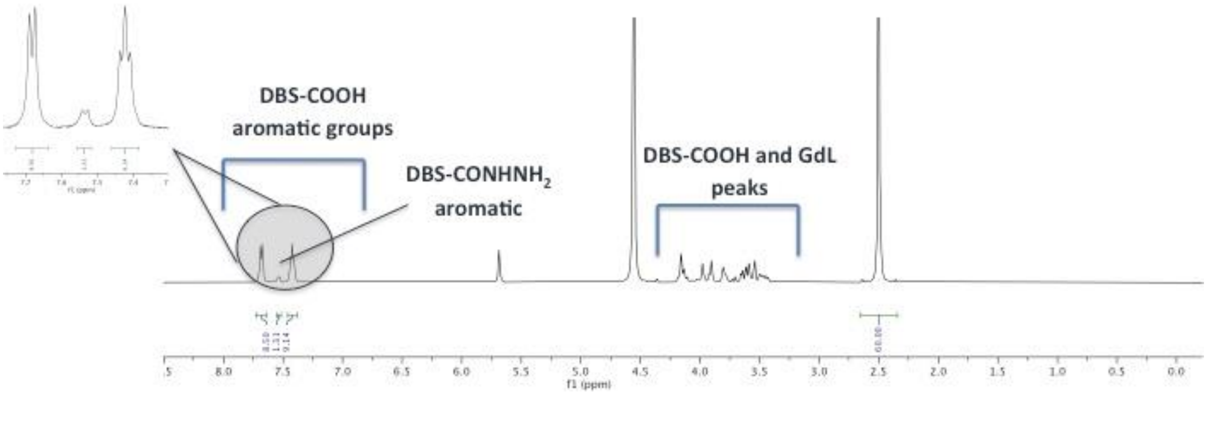

Fig. S28. ^{1}H NMR of outer domain of DBS-CONHNH$_2$/DBS-COOH gel prepared in a petri dish, 1h after the addition of GdL (0.07 mmol) to the reservoir in the middle.

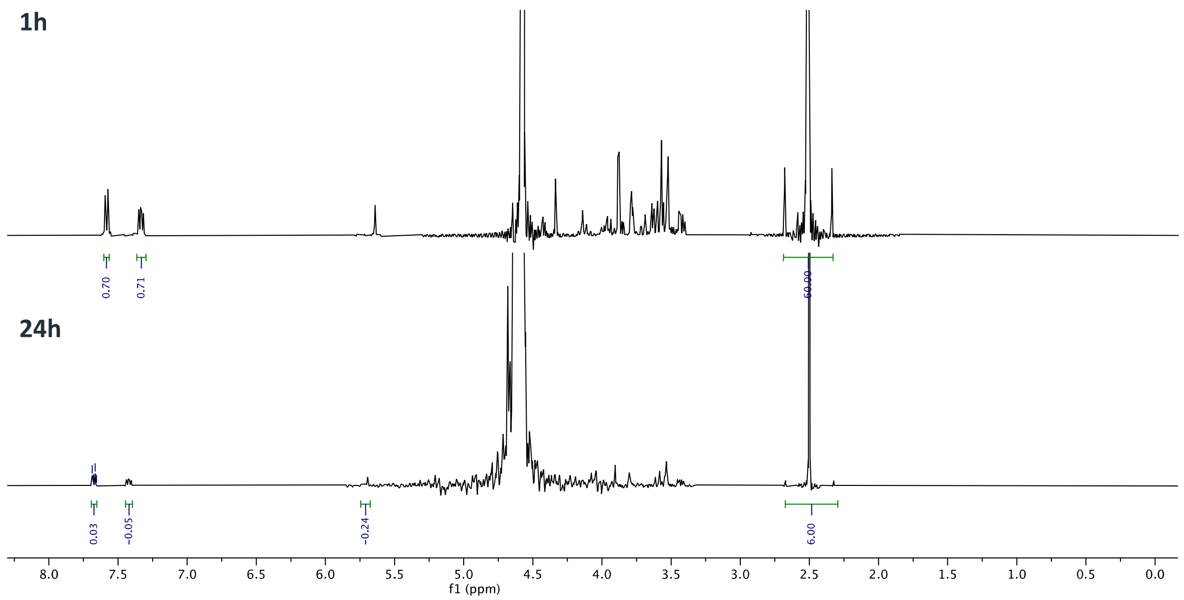

Fig. S29. ^{1}H NMR of inner domain of DBS-CONHNH$_2$/DBS-COOH gel prepared in a petri dish, 1 h and 24 h after the addition of GdL (0.07 mmol) to the reservoir in the middle.

**% of DBS-COOH**	**Time (hours)**			
	**1h**	**3h**	**6h**	**24h**
**Self-assembled**	45 %	77 %	87 %	97 %
**Non self-assembled**	55 %	23 %	13 %	3 %

Table S2. Percentage of self-assembled DBS-COOH in the DBS-COOH/DBS-CONHNH$_2$ two-component gel (inner domain) prepared with GdL (0.07 mmol), after 1, 3, 6 and 24 h.

S4.2 Quantification of self-assembled DBS-COOH by ^{1}H NMR (gels prepared in NMR tubes)

The DBS-CONHNH$_2$/DBS-COOH two-component gel sample used for this study was prepared in a 0.7 mL volume using a 0.4 % wt/vol concentration of each LMWG (0.006 mmol of DBS-CONHNH$_2$ and 0.006 mmol of DBS-COOH) and replacing water, NaOH (0.5 M, 4.2 μL) and HCl with the corresponding deuterated solutions. DMSO (1.4 μL) was added as an internal standard. The gel was formed into a NMR tube and DCl or GdL were added on top of each gel (each 0.02 mmol in 30 μL or 0.03 mmol in 30 μL). Spectra were recorded every hour for 12 hours and then after 24 and 48 hours. The concentration of the mobile components was calculated by comparison of the integrals of relevant peaks (DBS-COOH aromatic peak δ = 7.67) to that of DMSO (δ = 2.50 ppm).

To visually monitor the acid diffusion, the same gels were prepared in water in the presence of methylene blue (3 μL of a 1% EtOH solution) as an indicator. Colour changes due to acid diffusion through the gels were monitored at regular time intervals (1, 5, 10, 15, 30 min and 1, 1.5, 2, 4, 8, 24 and 48 h).

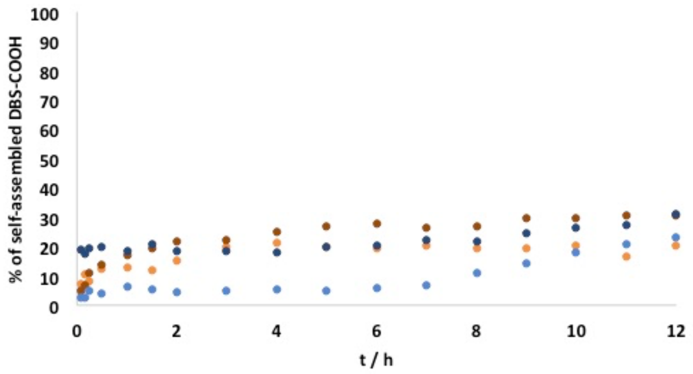

Fig. S30. Percentage of self-assembled DBS-COOH in DBS-CONHNH$_2$/DBS-COOH gel prepared in a NMR tube by addition of HCl (0.02 and 0.03 mmol, respectively light and dark blue dots) or GdL (0.02 and 0.03 mmol, respectively orange and brown dots). These values were calculated from the aromatic signal integrals of DBS-COOH in ^{1}H NMR spectra recorded every hour for 12 hours after acid addition.

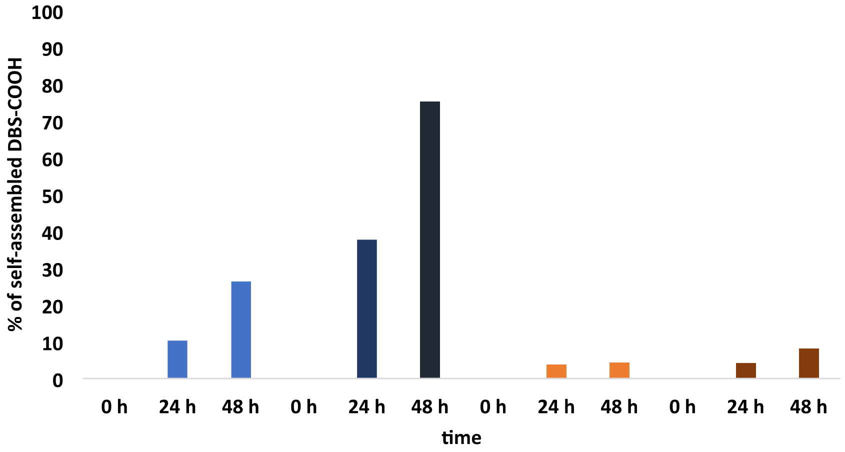

Fig. S31. Percentage of self-assembled DBS-COOH in DBS-CONHNH$_2$/DBS-COOH gel prepared in a NMR tube by addition of HCl (0.02 and 0.03 mmol, respectively light and dark blue bars) or GdL (0.02 and 0.03 mmol, respectively orange and brown bars). These values were calculated from the aromatic signal integrals of DBS-COOH in ^{1}H NMR spectra recorded after 24 and 48 hours from acid addition.

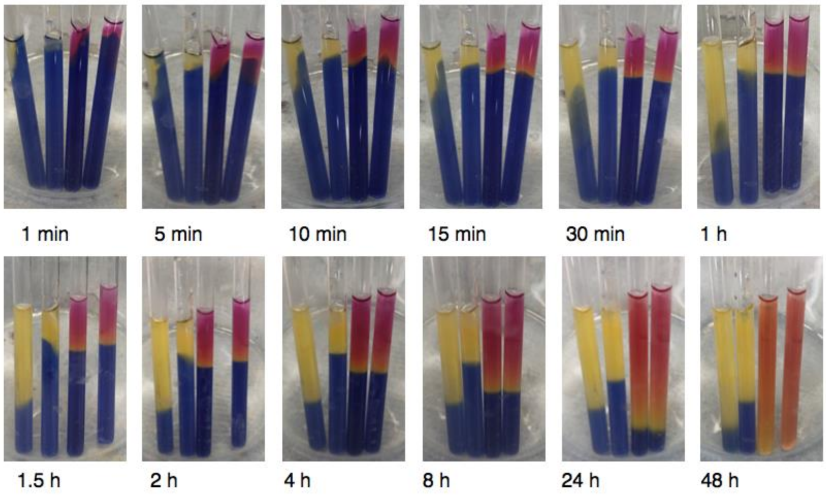

Figure S32. Photographs of gels prepared in NMR tubes with the thymol blue indicator after addition of: (from left to right) GdL (0.02 and 0.03 mmol, each 30 µL) and HCl (0.02 and 0.03 mmol, each 30 µL).

S5. Infrared (IR) Spectroscopy

S5.1 Preparation of xerogels

All the gels for IR were prepared in circular petri dishes (3 cm diameter) in a 2 mL total volume, by applying the method described in Sections S2.1 and S2.2. Once the gels were formed, an acid reservoir was cut in the middle using a 200 µL pipette tip and filled with HCl (0.012 mmol). The acid was left to diffuse through the gel and after 2 h, aliquots of the inner and outer part of the gel were collected and dried under vacuum. The resulting xerogels were placed into the infrared spectrophotometer and the spectra recorded in the range of 450-4000 cm^{-1}.

S5.2 IR spectra

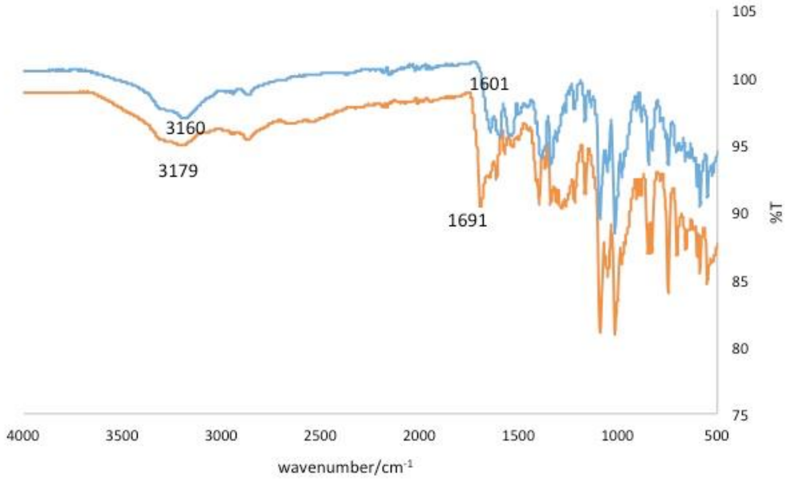

Figure S33. IR spectra of xerogels obtained from the inner (orange line) and outer (blue line) domains of a DBS-CONHNH$_2$/DBS-COOH gel (0.4 % wt/vol of each LMWG).

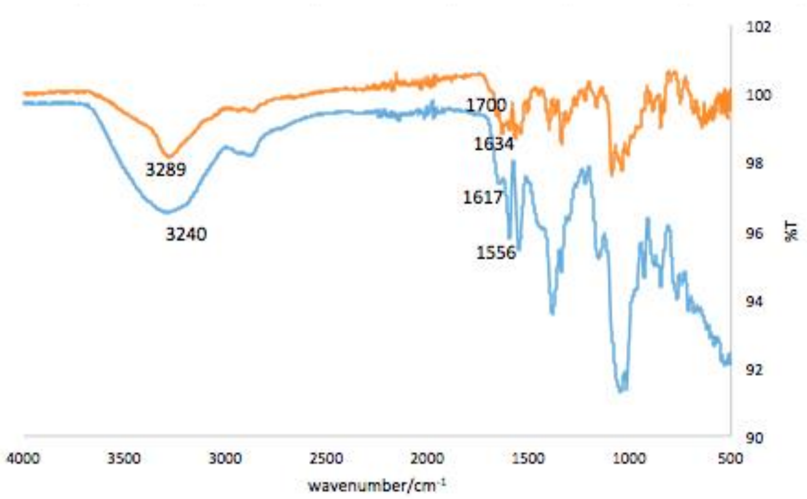

Figure S34. IR spectra of xerogels obtained from the inner (orange line) and outer (blue line) domains of a DBS-CONHNH$_2$/agarose/DBS-COOH gel prepared using 0.4 % wt/vol of each LMWG and 1.0% of agarose.

S6. Transmission and Scanning Electron Microscopy (TEM and SEM)

All the gels for microscopy were prepared in petri dishes (3 cm diameter) in a 2 mL volume. An acid reservoir was cut in the middle using a 200 μL pipette tip and filled with GdL (0.056 mmol). After 2 h, portions of the inner and outer domain were collected and prepared for the analysis. Control gels were also prepared in vials (1 mL volume) with or without GdL.

S6.1 Preparation of samples for TEM

Samples for TEM were obtained by adding a small amount of each sample on a copper grid. The excess of sample was removed with filter paper and allowed to set for 5 minutes. A negative stain (1 % uranyl acetate) was then added and the samples were left to rest for 30 minutes before taking the images.

S6.2 Preparation of samples for SEM

Samples for SEM were obtained by freeze drying the gels on copper shim pieces. The freeze-dried samples were then mounted on stubs and the images recorded.

S6.3 TEM and SEM images

S.6.3.1 TEM images of gel fibres

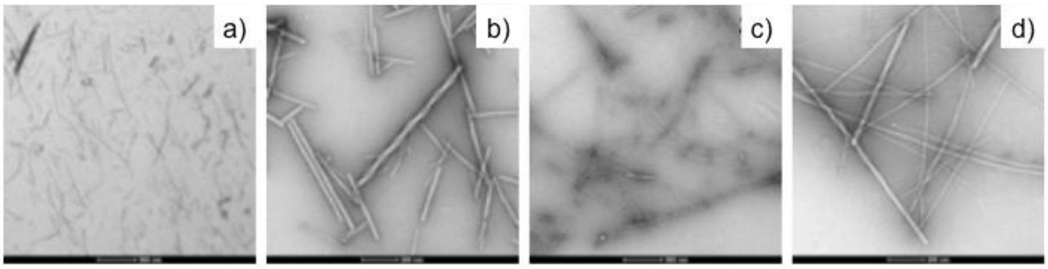

Figure S35. TEM images of DBS-CONHNH$_2$/DBS-COOH inner (a and b) and outer (c and d) gel domains prepared in petri dishes by GdL diffusion (0.056 mmol). Scale bars: 500 nm (a and c) and 200 nm (b and d).

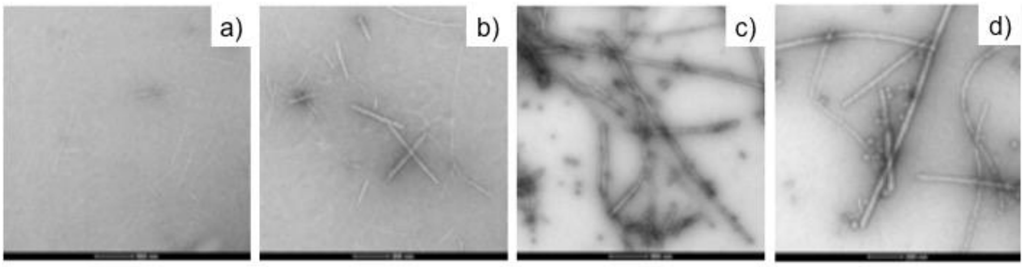

Figure S36. TEM images of DBS-CONHNH$_2$/DBS-COOH gels in vials prepared with (a and b) and without GdL (c and d). Scale bars: 500 nm (a and c) and 200 nm (b and d).

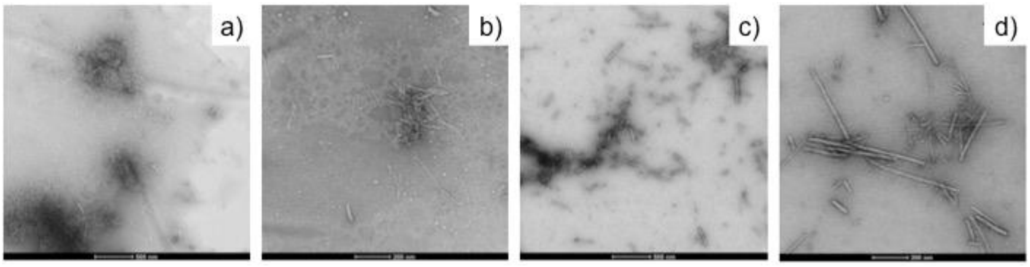

Figure S37. TEM images of DBS-CONHNH$_2$/agarose/DBS-COOH inner (a and b) and outer (c and d) gel domains prepared in petri dishes by GdL diffusion (0.056 mmol). Scale bars: 500 nm (a and c) and 200 nm (b and d).

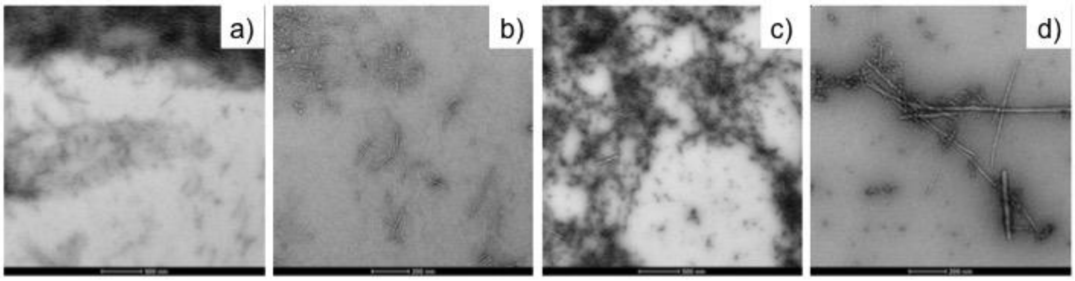

Figure S38. TEM images of DBS-CONHNH$_2$/agarose/DBS-COOH gels in vials prepared with (a and b) and without GdL (c and d). Scale bars: 500 nm (a and c) and 200 nm (b and d).

S.6.3.2 Fibre size measurements

The fibre width of the gel networks was measured in the TEM images using the *ImageJ* Software. For each gel, 25 fibres were measured and the obtained values were reported in the graphs below.

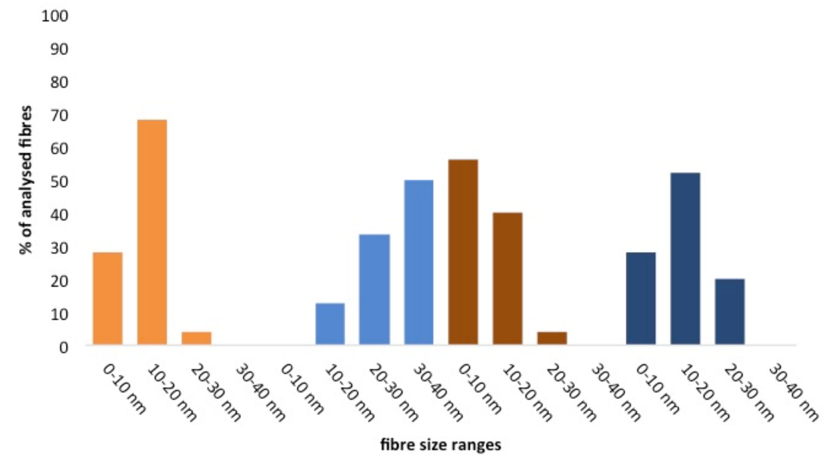

Figure S39. Fibre widths of DBS-CONHNH$_2$/DBS-COOH gels prepared in vials with and without GdL (respectively orange and light blue bars), and DBS-CONHNH$_2$/DBS-COOH inner and outer gel domains (respectively brown and dark blue bars) prepared in petri dishes by GdL diffusion (0.056 mmol).

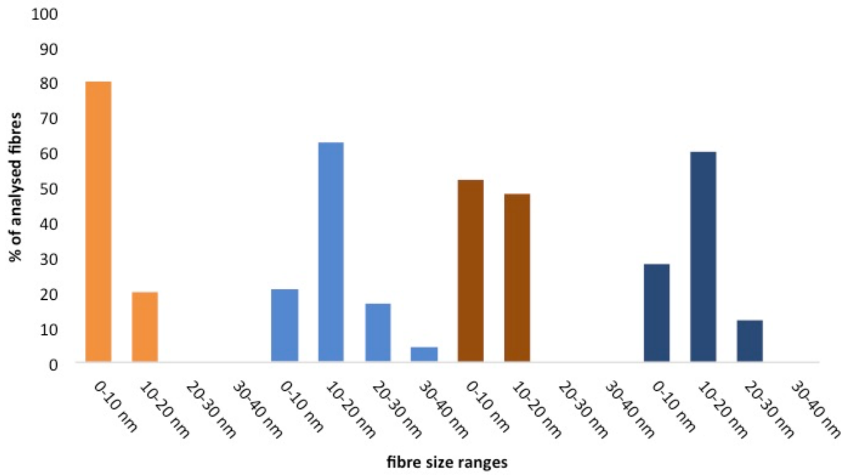

Figure S40. Fibre widths of DBS-CONHNH$_2$/agarose/DBS-COOH gels prepared in vials with and without GdL (respectively orange and light blue bars), and DBS-CONHNH$_2$/agarose/DBS-COOH inner and outer gel domains (respectively brown and dark blue bars) prepared in petri dishes by GdL diffusion (0.056 mmol).

S.6.3.1 SEM images of gel fibres

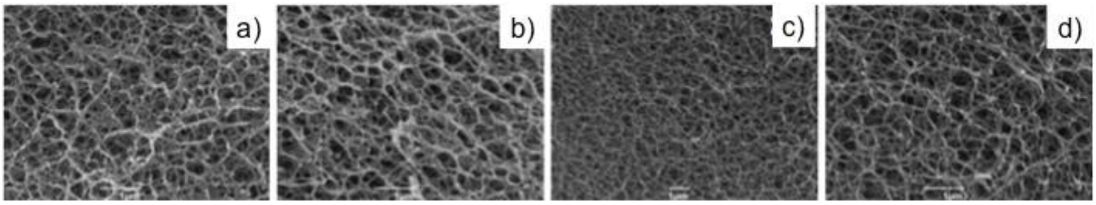

Figure S41. SEM images of DBS-CONHNH$_2$/DBS-COOH inner (a and b) and outer (c and d) gel domains prepared in petri dishes by GdL diffusion (0.056 mmol). Scale bars 1 μm.

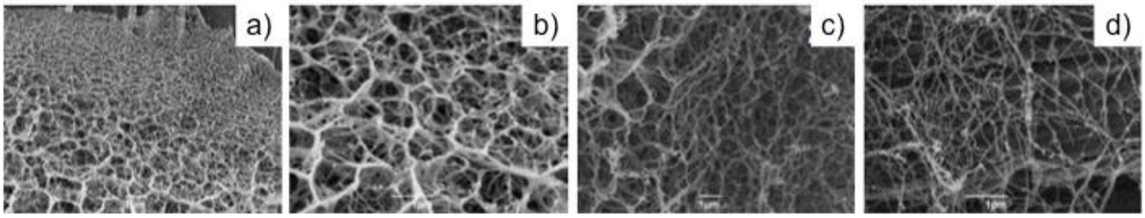

Figure S42. SEM images of DBS-CONHNH$_2$/DBS-COOH gels prepared in vials with (a and b) and without GdL (c and d). Scale bars 1 μm.

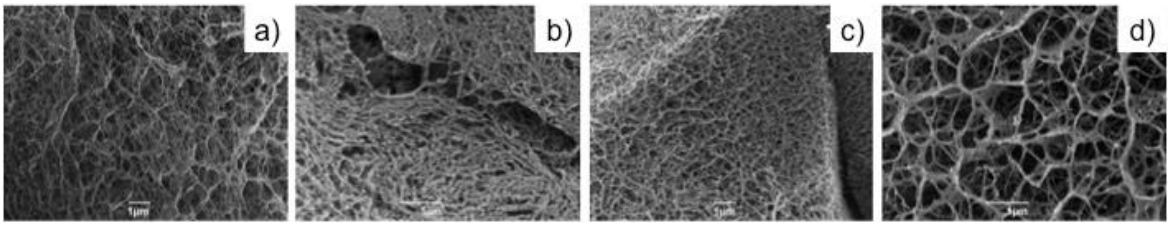

Figure S43. SEM images of DBS-CONHNH$_2$/agarose/DBS-COOH inner (a and b) and outer (c and d) gel domains prepared in petri dishes by GdL diffusion (0.056 mmol). Scale bars 1 μm.

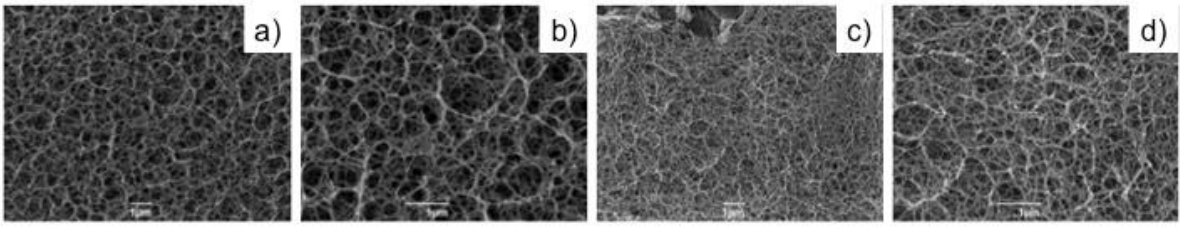

Figure S44. SEM images of DBS-CONHNH$_2$/agarose/DBS-COOH gels prepared in vials with (a and b) and without GdL (c and d). Scale bars 1 μm.

S7. Rheology

S7.1 Methodology

Gel samples for rheology were prepared in circular petri dishes (8 cm diameter; 6 mL total volume) with HCl (0.13 mmol, 35 μL) being loaded into a central reservoir. Samples were cut from inner and outer domains of the gel using a bottomless vial (2 cm diameter), carefully transferred to the rheometer and analysed. Gels in bottomless vials (1 mL volume) were also prepared for comparison using GdL (0.056 mmol). The measurements were carried out at 25 °C using a 2 cm parallel plate and a gap of 0.5 mm for the HCl gels and 2 mm for the GdL gels. To avoid solvent evaporation and keep the sample hydrated, a solvent trap was used, and the internal atmosphere was kept saturated. Amplitude sweep experiments were performed in the range of 0.05-100% strain at a 1 Hz frequency to identify the linear viscoelastic region. Frequency sweep experiments were performed between 0.1 and 100 Hz using a shear strain of 0.25%. The measurements were repeated three times to ensure reproducibility and the average data are shown. It is important to note that in our frequency sweep experiments we extend to much higher frequencies than in most reported cases. This leads us to see a frequency induced change in rheological behaviour. Other researchers have observed similar changes at very high frequencies.[3]

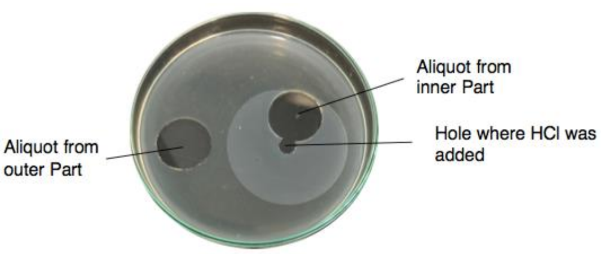

Figure S45. Example of DBS-CONHNH$_2$/agarose/DBS-COOH gel for rheology prepared in an 8 cm diameter petri dish. Aliquots of the inner and outer domains were cut using bottomless vials and transferred to the rheometer.

S7.2 Rheology data

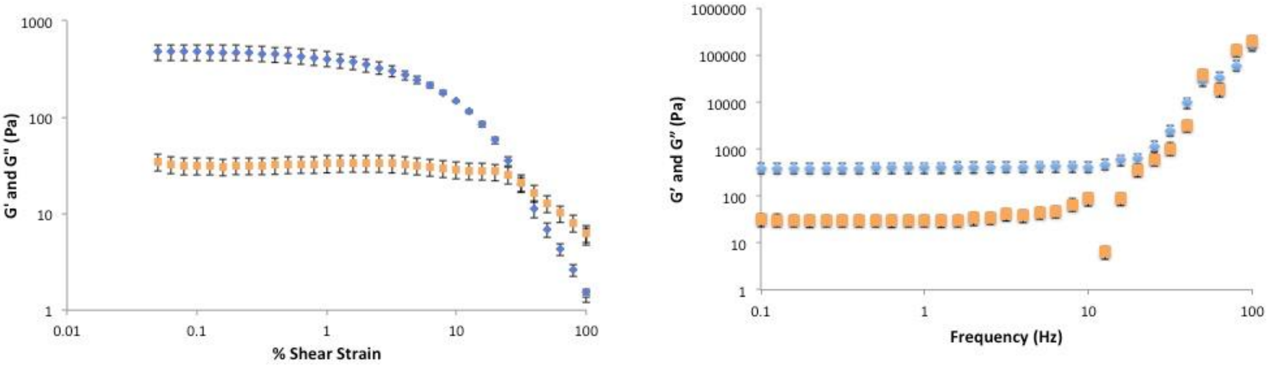

Figure S46. Elastic (G', blue circles) and viscous (G", orange circles) moduli of DBS-COOH hydrogel prepared in vial (0.4 % wt/vol) with increasing shear strain (left) and frequency (right).

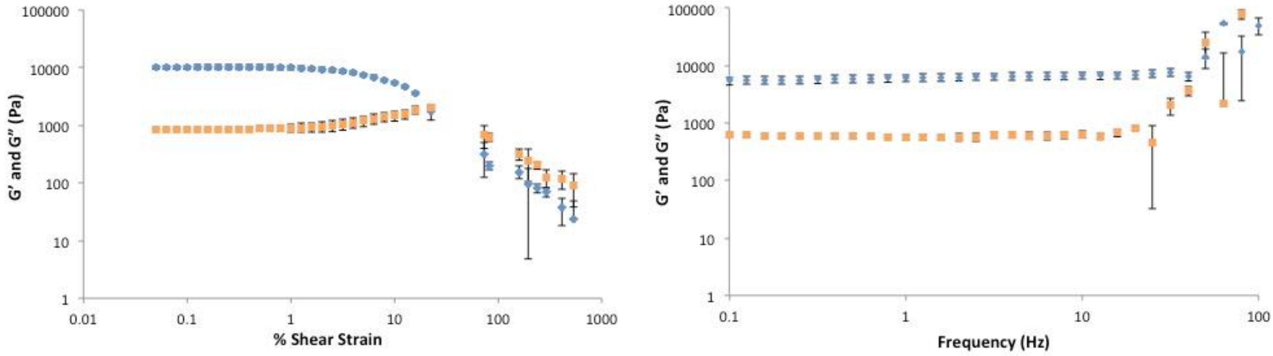

Figure S47. Elastic (G', blue circles) and viscous (G", orange circles) moduli of DBS-COOH/agarose hydrogel (prepared in vial with 0.4 % wt/vol DBS-COOH and 1.0 % wt/vol agarose) with increasing shear strain (left) and frequency (right).

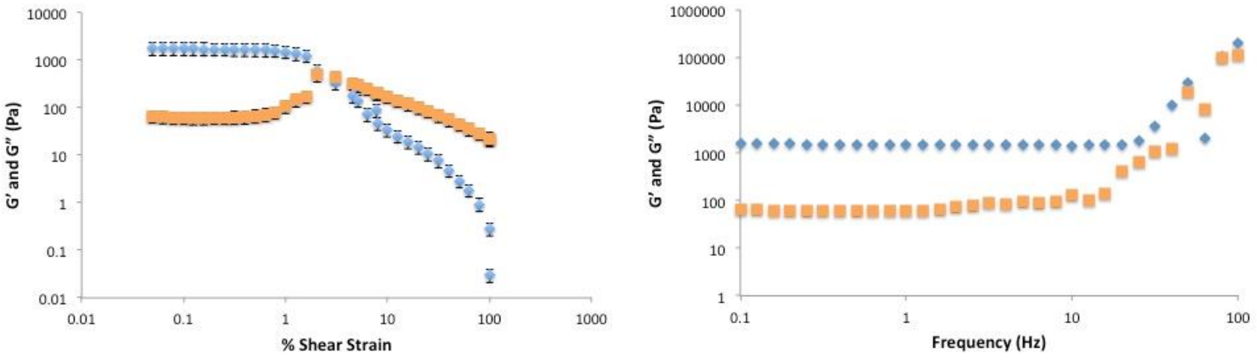

Figure S48. Elastic (G’, blue circles) and viscous (G”, orange circles) moduli of DBS-CONHNH$_2$ hydrogel prepared in vial (0.4 % wt/vol) with increasing shear strain (left) and frequency (right).

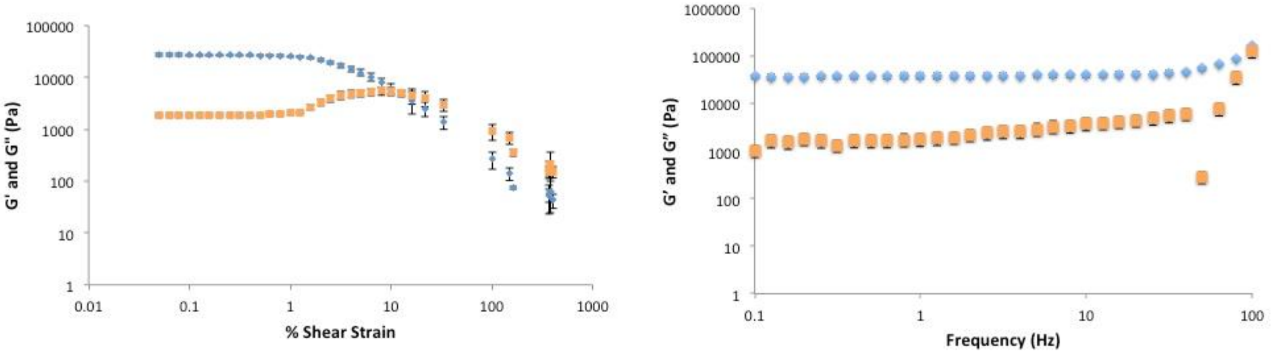

Figure S49. Elastic (G’, blue circles) and viscous (G”, orange circles) moduli of DBS-CONHNH$_2$/agarose hydrogel (prepared in vial with 0.4 % wt/vol DBS-CONHNH$_2$ and 1.0 % wt/vol agarose) with increasing shear strain (left) and frequency (right).

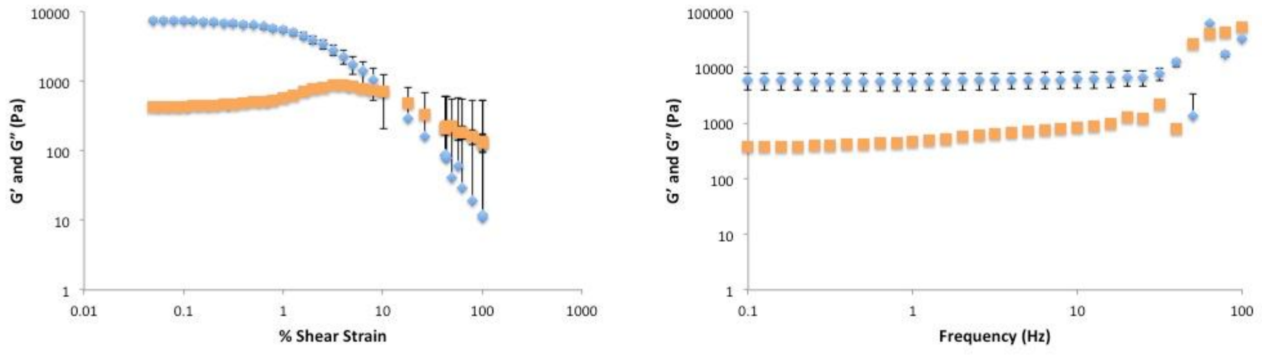

Figure S50. Elastic (G’, blue circles) and viscous (G”, orange circles) moduli of DBS-CONHNH$_2$/DBS-COOH gel (prepared in vial with 0.4 % wt/vol of each LMWG) with increasing shear strain (left) and frequency (right).

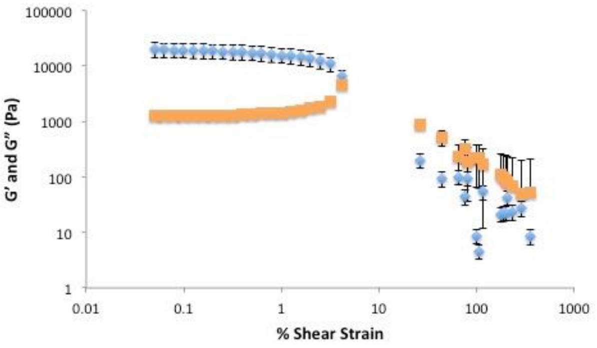

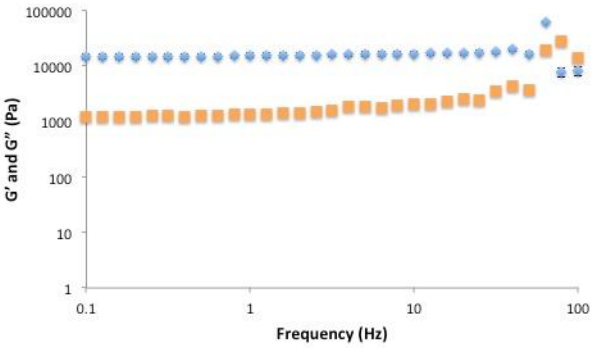

Figure S51. Elastic (G', blue circles) and viscous (G", orange circles) moduli of DBS-CONHNH$_2$/agarose/DBS-COOH gel (prepared in vial with 0.4 % wt/vol of each LMWG and 1.0 % wt/vol agarose) with increasing shear strain (left) and frequency (right).

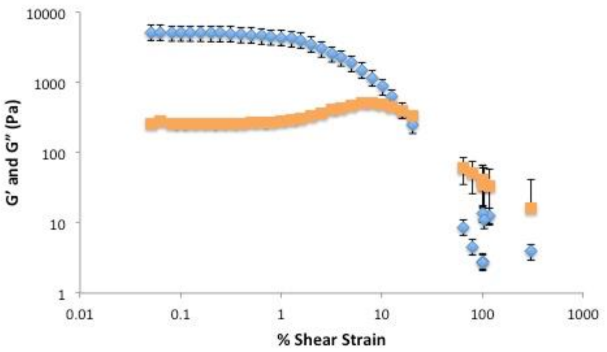

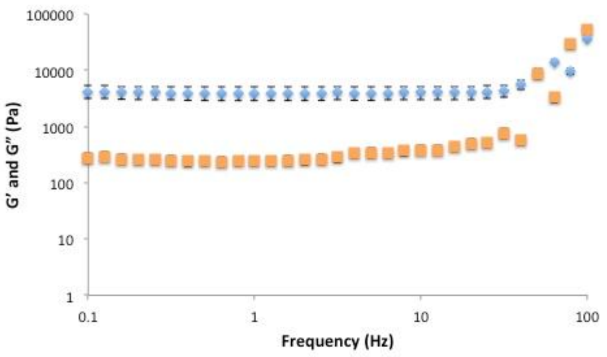

Figure S52. Elastic (G', blue circles) and viscous (G", orange circles) moduli of DBS-CONHNH$_2$/DBS-COOH inner domain gel (prepared in a petri dish with 0.4 % wt/vol of each LMWG) with increasing shear strain (left) and frequency (right).

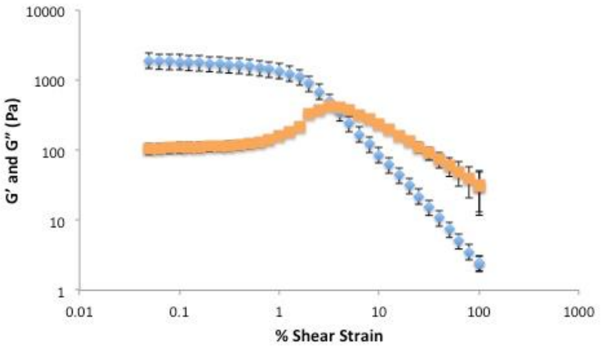

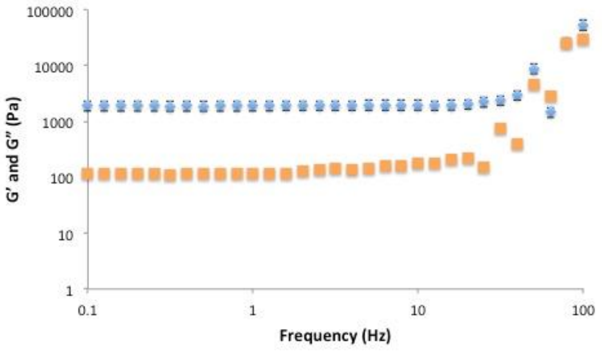

Figure S53. Elastic (G', blue circles) and viscous (G", orange circles) moduli of DBS-CONHNH$_2$/DBS-COOH outer domain gel (prepared in a petri dish with 0.4 % wt/vol of each LMWG) with increasing shear strain (left) and frequency (right).

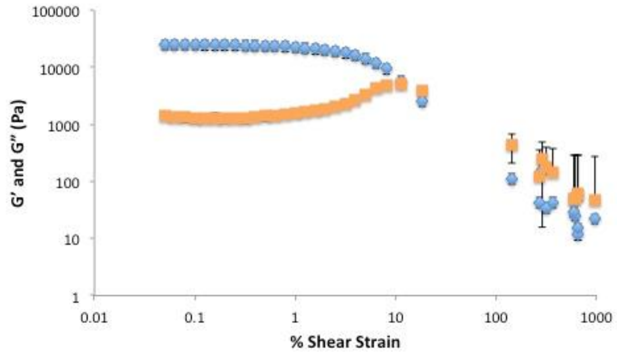

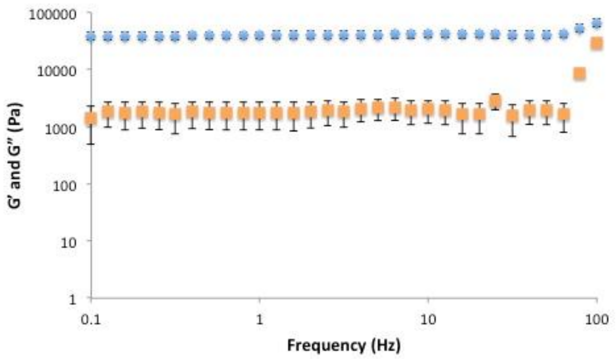

Figure S54. Elastic (G', blue circles) and viscous (G", orange circles) moduli of DBS-CONHNH$_2$/agarose/DBS-COOH inner domain gel (prepared in a petri dish with 0.4 % wt/vol of each LMWG and 1.0% wt/vol agarose) with increasing shear strain (left) and frequency (right).

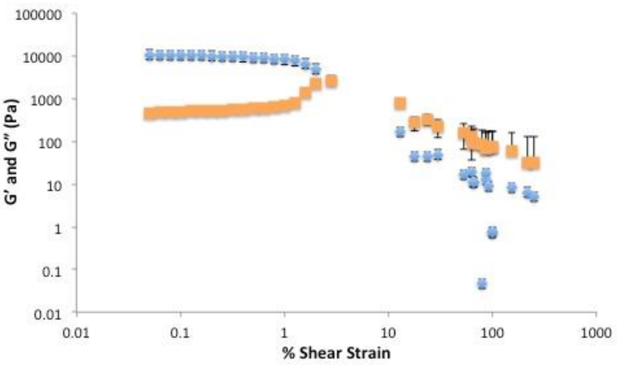

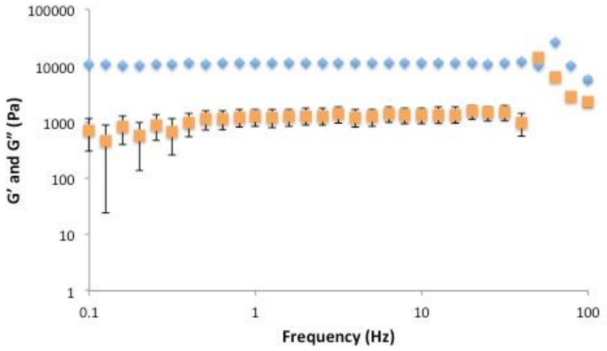

Figure S55. Elastic (G', blue circles) and viscous (G", orange circles) moduli of DBS-CONHNH$_2$/agarose/DBS-COOH outer domain gel (prepared in a petri dish with 0.4 % wt/vol of each LMWG and 1.0% wt/vol agarose) with increasing shear strain (left) and frequency (right).

S7.3 Temporal rheology study

A temporal study was performed on DBS-CONHNH$_2$/agarose/DBS-COOH gels prepared in circular petri dishes (6 mL total volume) with a diameter of 8 cm, with HCl (0.13 mmol, 35 μL) being loaded into a central reservoir. Samples were cut from inner domain of the gel using a bottomless vial (2 cm diameter) after 1, 2 and 6 hours, carefully transferred to the rheometer and analysed.

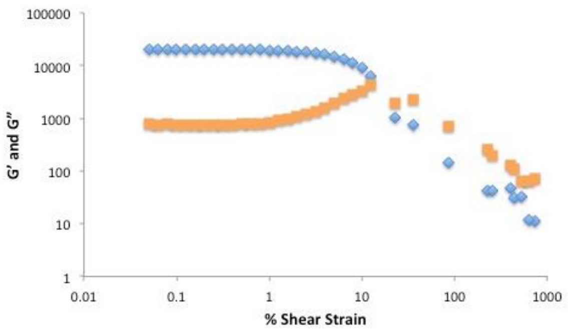

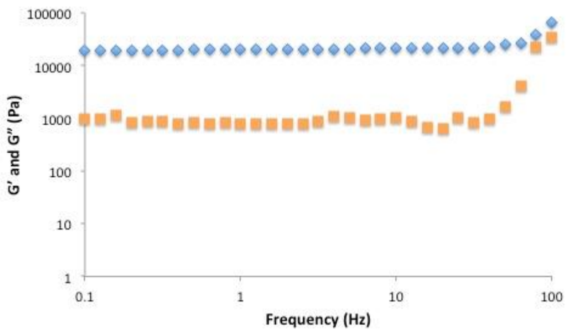

Figure S56. Elastic (G', blue circles) and viscous (G", orange circles) moduli of DBS-CONHNH$_2$/agarose/DBS-COOH inner domain gel (prepared in a petri dish with 0.4 % wt/vol of each LMWG and 1.0% wt/vol agarose) after 1h with increasing shear strain (left) and frequency (right).

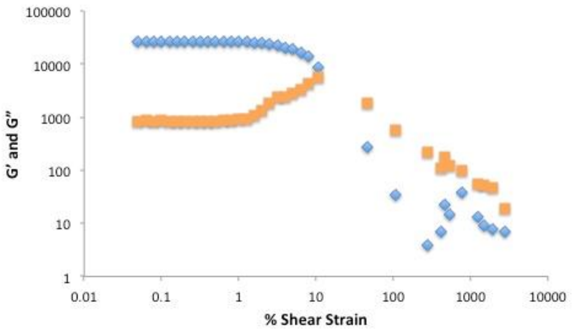

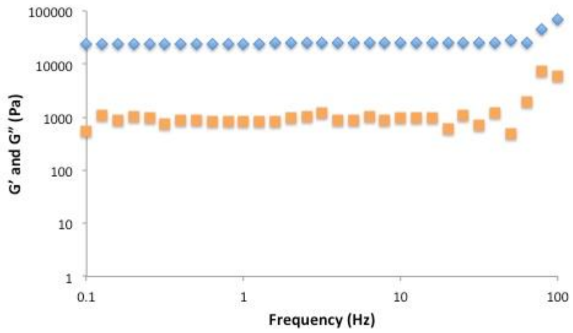

Figure S57. Elastic (G', blue circles) and viscous (G", orange circles) moduli of DBS-CONHNH$_2$/agarose/DBS-COOH inner domain gel (prepared in a petri dish with 0.4 % wt/vol of each LMWG and 1.0% wt/vol agarose) after 2h with increasing shear strain (left) and frequency (right).

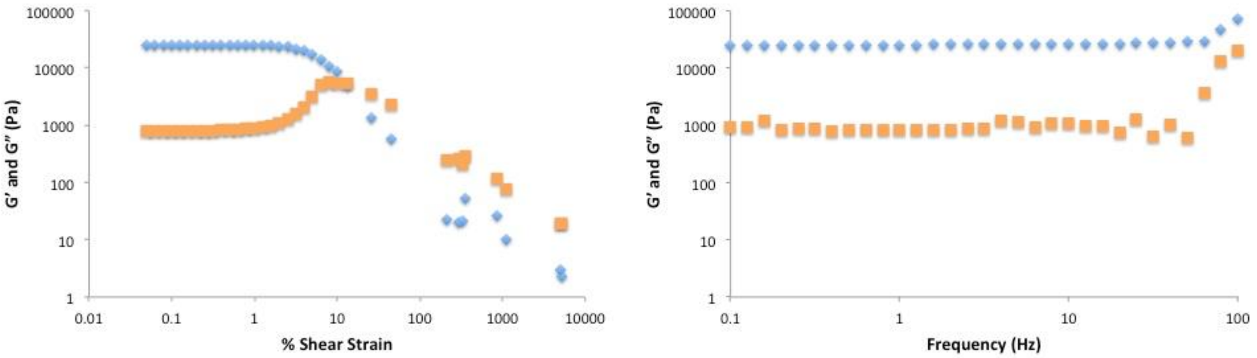

Figure S58. Elastic (G', blue circles) and viscous (G", orange circles) moduli of DBS-CONHNH$_2$/agarose/DBS-COOH inner domain gel (prepared in a petri dish with 0.4 % wt/vol of each LMWG and 1.0% wt/vol agarose) after 6h with increasing shear strain (left) and frequency (right).

S8. References

1. Okesola, B. O.; Smith, D. K., *Chem. Commun.* 2013**, 49**, 11164-11166.
2. Cornwell, D. J.; Okesola, B. O.; Smith, D. K., *Soft Matter* 2013**, 9**, 8730-8736.
3. (*a*) E. R. Draper, L. L. E. Mears, A. M. Castilla, S. M. King, T. O. McDonald, R. Akhtar and D. J. Adams, *RSC Adv.*, 2015, **5**, 95369–95378; (*b*) Y. Abidine, V. M. Laurent, R. Michel, A. Duperray, L. I. Palade and C. Verdier, *Europhys. Lett.*, 2015, **109**, 38003.
